# A Simplified Guide RNA Synthesis Protocol for SNAP- and Halo-Tag-Based RNA Editing Tools

**DOI:** 10.3390/molecules30051049

**Published:** 2025-02-26

**Authors:** Daniel Tobias Hofacker, Sebastian Kalkuhl, Jana Franziska Schmid, Shubhangi Singh, Thorsten Stafforst

**Affiliations:** 1 Interfaculty Institute of Biochemistry, University of Tübingen, 72076 Tübingen, Germany; 2 Gene and RNA Therapy Center (GRTC), Faculty of Medicine, University Tübingen, 72076 Tübingen, Germany

**Keywords:** RNA base editing, SNAP-tag, Halo-tag, guide RNA, protocol

## Abstract

SNAP-tag and Halo-tag have been employed to achieve targeted RNA editing by directing the deaminase domain of human ADAR to specific sites in the transcriptome. This targeting is facilitated by short guide RNAs (gRNAs) complementary to the target transcript, which are chemically modified with benzylguanine or chloroalkane moieties to enable covalent binding to the respective self-labeling enzymes. However, broad application of this approach has been limited by challenges such as low scalability, the requirement for specialized chemical expertise and equipment, and labor-intensive protocols. In this study, we introduce streamlined, efficient protocols for the synthesis and purification of these linkers, suitable for SNAP-tag and Halo-tag applications, without the need for advanced chemical equipment. Our methods enable linker coupling in a kit-like manner and support the high-yield production of modified gRNAs. We demonstrate that the newly synthesized linkers and gRNA designs perform similarly to previously published constructs with regard to RNA editing efficiency. Moreover, large-scale production of modified gRNAs facilitates their use in studies involving cellular uptake and in vivo experiments.

## 1. Introduction

**Figure 1:**
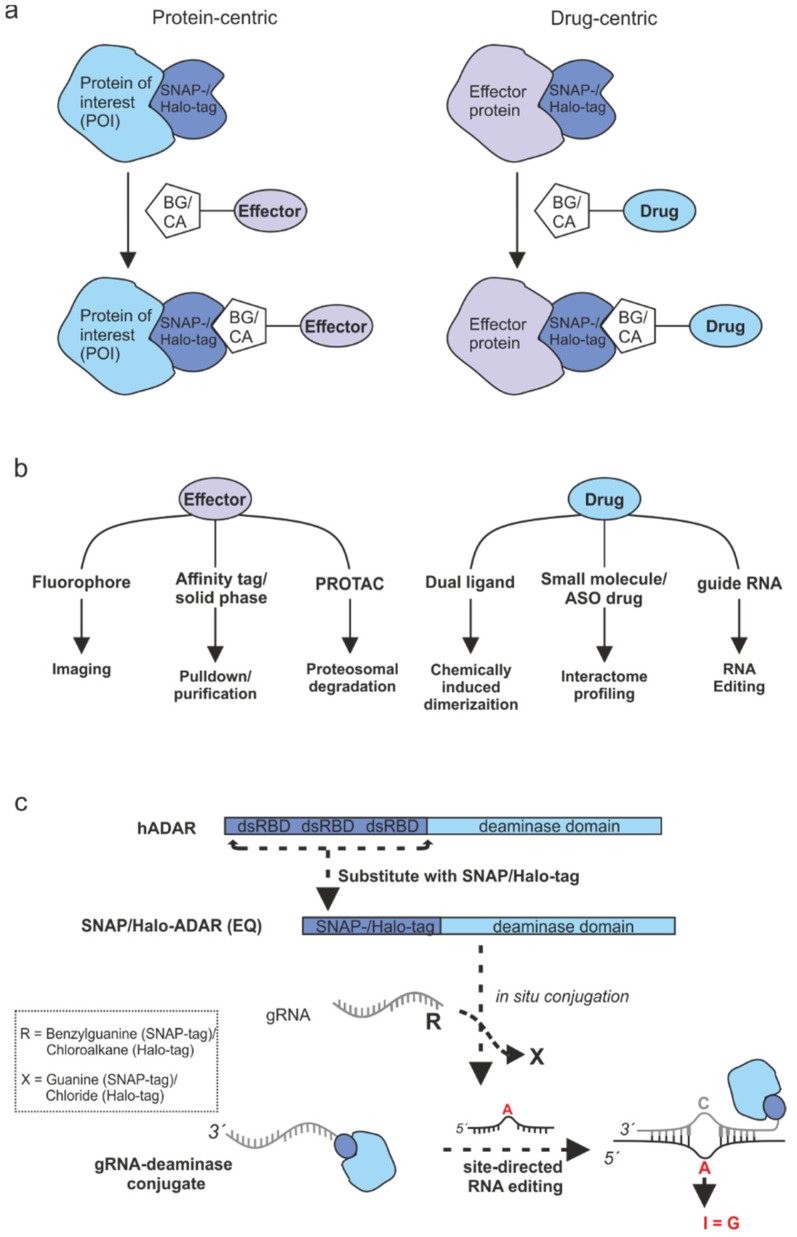
Self-labeling enzymes have various applications. (**a**) Various effectors are covalently conjugated to SNAP-tagged/Halo-tagged fusion proteins by using the self-labeling moieties benzylguanine (BG) or chloroalkane (CA), respectively. In protein-centric assays (left panel), an effector molecule is recruited to a protein of interest (POI). In drug-centric assays (right panel), an effector protein is recruited to a drug-of-interest (DOI). (**b**) Schematic representation of different applications of self-labeling enzyme tags in protein-centric (left panel) and drug-centric (right panel) approaches. (**c**) Detailed scheme representing SNAP-tag/Halo-tag-based RNA editing platforms. ADAR’s double-stranded RNA binding domains (dsRBD) were replaced by a SNAP-tag or Halo-tag to allow for in situ conjugation of guide RNA and deaminase. Hybridization of the guide RNA to the target RNA controls precise and efficient A-to-I RNA base editing. PROTAC = proteasomal targeting chimeras, ASO = antisense oligonucleotide.

Self-labeling enzyme tags apply single-turnover suicide reactions to irreversibly transfer a self-labeling moiety covalently to themselves. This allows for modifying fusion proteins of self-labeling tags with dyes, small molecules, chemically induced dimerizers, and/or nucleic acids in a defined stoichiometry, and has been used extensively for super-resolution microscopy [1], manipulation of protein function [2,3,4], assembly of higher-ordered structures [5,6,7,8], target identification [9,10], or oligonucleotide-guided effectors [11,12] (Figure 1a). The first self-labeling enzyme was the SNAP-tag, which was engineered in 2003 from human *O^6^*-alkylguanine-DNA-alkyltransferase (hAGT) to accept the non-natural self-labeling moiety *O^6^*-benzylguanine (BG) with high efficiency and specificity [13]. Later, the CLIP-tag [14] and the Halo-tag were reported, with the latter being engineered from a bacterial haloalkane dehalogenase to accept the chloroalkane (CA) self-labeling moiety [15]. Besides their respective self-labeling moieties, the different self-labeling enzymes differ in their reaction kinetics, size, and charge [16], but have all been demonstrated to be applicable in vitro, in cell culture, but also in vivo [17,18]. To a certain extent, self-labeling enzymes can be used side-by-side orthogonally inside the same cell, however, low-level cross-reactivity between SNAP- and CLIP-tag substrates suggest that the combination of SNAP- and Halo-tag is better suitable in such cases [14,16,19].

The versatility of such self-labeling enzyme tags has been demonstrated in numerous cases including imaging studies in fixed cells, living cells, and microorganisms, monitoring protein/drug delivery, protein purification, protein structure analysis, and recruitment of effector domains (Figure 1b), as reviewed in refs. [20,21,22]. The first applications were mainly based on fluorescent imaging and protein localization studies and were later extended by photoactivatable probes and interaction studies based on fluorescence and bioluminescence resonance energy transfer (FRET/BRET) [6,23,24]. Further, through the immobilization of tagged fusion proteins on beads, microassays for studying protein–protein and protein–DNA interactions have been developed [25]. By attaching small molecules to the respective self-labeling moiety, the recruitment of effector molecules or effector proteins to the protein of interest (POI) enables the manipulation of the latter. Typical examples are the chemically induced dimerization of proteins under photocontrol [26,27] or proteasomal degradation of the POI [28,29]. Recently, SNAP-tag and Halo-tag were applied to detect the protein interactome of small-molecule drugs and RNA drugs inside the living cell by covalent recruitment of a promiscuous biotin ligase to the respective drug moiety [30].

In our lab, we pioneered the application of self-labeling enzyme tags for the assembly of engineered (guide)RNA-protein conjugates (Figure 1c). Specifically, we used a fusion of the SNAP-tag with the deaminase domain of human adenosine deaminase acting on RNA (ADAR) to engineer an effector that allows for programmable adenosine-to-inosine (A-to-I) RNA base editing [12] by covalently attaching a chemically stabilized, short guide RNA in situ to the SNAP-tagged deaminase [12,19,31]. Later, we demonstrated orthogonal A-to-I and cytidine-to-uridine (C-to-U) editing by combining a SNAP-tagged ADAR deaminase domain with a Halo-tagged RESCUE-S domain within the same cell [19]. Most recently, we developed a new guide RNA design that is based on the recruitment of two SNAP-ADAR effectors per guide RNA and that achieves highly efficient and precise RNA base editing with extended codon scope in various cell lines, including primary cells [19,31,32]. Finally, photocaged self-labeling moieties have been applied in cell culture and in vivo to control RNA base editing by light [33]. The incorporation of noncanonical nucleobases and backbone modifications into the guide RNA component resulted in clear advantages of the approach. Nevertheless, competing approaches that rely on easy-to-clone, fully genetically encoded alternatives are typically applied more widely [34]. We identified the cumbersome protocols for the synthesis of mono- and bivalent self-labeling moieties and its attachment to purchased guide RNAs as the limiting factor. Indeed, our current protocols require manual solid phase synthesis and HPLC purification of the self-labeling moiety, and urea PAGE-purification of the guide RNA after the attachment reaction [19,35].

Here, we now provide a simplified protocol, which uses only very simple preparative organic chemistry and avoids any cumbersome purification steps, like silica gel chromatography, HPLC, or urea PAGE. This new protocol allows for using the SNAP- and Halo-ADAR approaches in a more kit-like fashion. It will make largerquantities of modified guide RNAs accessible and affordable that are required for uptake and in vivo studies.

## 2. Results

### 2.1. Simplified Synthesis Protocol of NHS-Activated Self-Labeling Moieties

**Figure 2:**
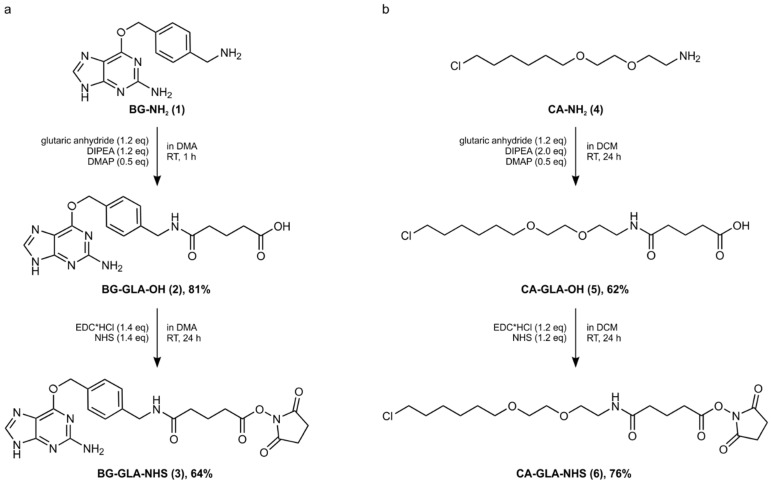
Synthesis of NHS-activated self-labeling moieties for BG and CA. (**a**) BG-GLA-NHS (**3**) was synthesized from commercially available BG-NH_2_ (**1**) in two steps. (**b**) CA-GLA-NHS (**6**) was synthesized analogously from commercially available CA-NH_2_ (**4**). Both syntheses allow for fast and simple cleanup on the gram scale.

In previous protocols [12,19], we described the synthesis of rather complex linkers by manual solid phase organic synthesis followed by preparative HPLC. Such linkers contain either one (linear) or two (branched) self-labeling moieties, e.g., BG or CA, respectively, attached to a polyethylene glycol linker with a terminal carboxylic acid (Supplementary Figure S1). To install the self-labeling moiety onto a guide RNA of choice, the carboxylic acid was activated as an *N*-hydroxy succinimide (NHS) ester in situ to react with an amino linker on the commercially available guide RNA. This conjugation reaction was prone to side reactions and typically required the purification of the product guide RNA from unreacted, starting guide RNA by urea PAGE purification, followed by gel extraction and ethanol precipitation. To address the low scalability of solid phase synthesis, we now established two simple, liquid phase, preparative synthesis protocols that make the amino-reactive NHS esters directly accessible for both moieties, BG and CA. By slightly modifying a published synthesis route [36] and by optimizing precipitation and washing protocols, we were able to obtain BG-GLA-NHS (**3**) in two steps from commercially available BG-NH_2_ (**1**), as shown in Figure 2a. Importantly, both steps can be carried out with simple equipment and on a gram scale, as described in detail in Section 4. The overall yield of the two steps was 51%. The focus was put on the simplicity of the procedure rather than optimizing yield or purity. The compound was purified by precipitation and washing of the precipitate in both synthesis steps. The integrity of the intermediate and the final product **3** were confirmed by HPLC (Supplementary Figures S2 and S3), ^1^H- and ^13^C-NMR, and high-resolution mass spectrometry (see the Section 4 and Supplementary Materials). The purity was sufficient for subsequent conjugation to a guide RNA. The synthesis of CA-GLA-NHS (**6**) was achieved via an analog two-step route with a slightly different synthesis and purification protocol, starting from CA-NH_2_ (**4**) (see Figure 2b and the Section 4 below). The overall yield was 47%. The compound was purified by liquid-liquid extraction in both synthesis steps. The integrity of the final product **6** was confirmed by ^1^H- and ^13^C-NMR and high-resolution mass spectrometry (see the Section 4). Both pre-activated NHS esters (**3** and **6**) have been used without further purification in the attachment protocol (see below) and can be stored for at least 6 months in dried format without loss in coupling performance.

### 2.2. Simplified Protocol for the Attachment of the Self-Labeling Moiety to the Guide RNA

**Figure 3:**
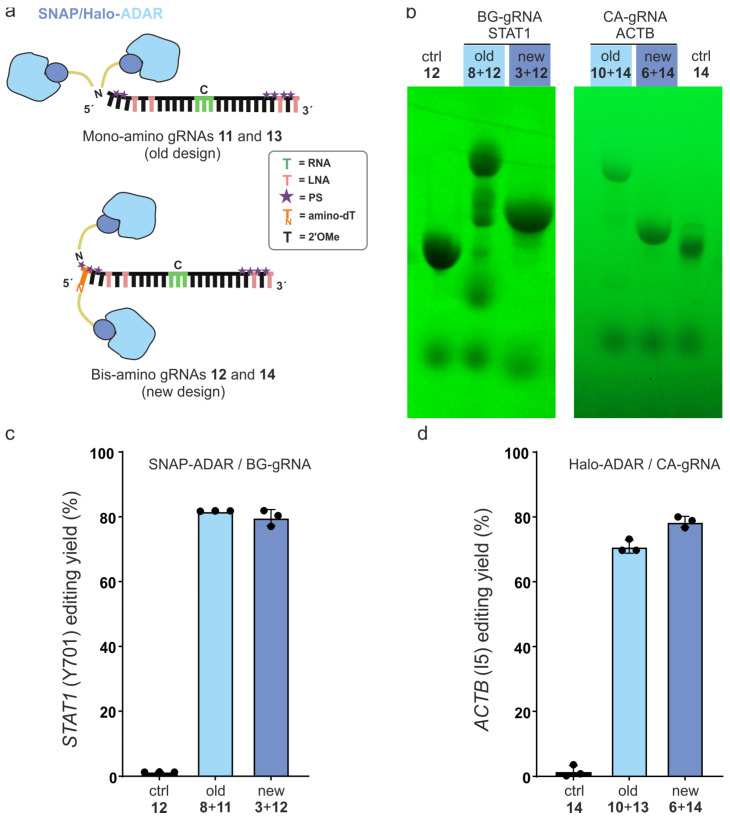
Benchmark of guide RNAs carrying two self-labeling moieties (BG or CA) generated with the new, simplified versus old protocol. (**a**) In the old protocol, a complex branched linker, derived from NHS ester **8** (BG) or **10** (CA), was coupled to mono-amino guide RNAs carrying one 5′-terminal amino linker, e.g., gRNA **11** and **13**. In the new protocol, two linear linkers, derived from simple NHS esters **3** (BG) or **6** (CA), are coupled to bis-amino guide RNAs like gRNA **12** and **14**, which carry an additional amino-modified dT nucleobase at their 5′-terminus, next to their 5′-amino linker. (**b**) Analytical urea PAGE shows increased turnover and purity of gRNAs prepared by the new protocol. (**c**) Editing performance of BG guide RNAs (generated via old or new protocol, respectively) targeting the endogenous STAT1 transcript (Tyr 701) in HeLa-PB-SNAP-ADAR1Q cells induced with 1 µg/mL doxycycline for 24 h. (**d**) Editing performance of CA guide RNAs (generated via old or new protocol, respectively) targeting the endogenous ACTB transcript (Ile 5) in 293 Flp-In Halo-ADAR1Q cells induced with 10 ng/mL doxycycline for 24 h. n = 3 independent experiments. Modifications: LNA = locked nucleic acid, PS = phosphorothioate, amino-dT = amino-C6-deoxythymidine, 2′OMe = 2′O-methyl.

Previously, we purchased gRNAs with a 5′-terminal C6 amino linker (Figure 3a) [19,35] and coupled pre-activated linear or branched linkers to them. Therefore, we pre-activated a large excess (~20-fold per guide RNA) of the respective, HPLC-purified, linkers (**7**–**10**, for structures see Supplementary Figure S1) with DIC/NHS in situ, with a typical yield of not more than 50% (Supplementary Figure S4). The respective NHS ester was then incubated for two hours at 37 °C with the purchased guide RNAs, like guide RNAs **11** and **13** (see Figure 3a) [19,35]. Typically, we observed incomplete turnover of the guide RNA, likely due to side reaction of the terminal amino linker of the guide RNA with the remaining coupling agent, as exemplified here for the reaction of branched BG linker **8** with guide RNA **11**, or branched CA linker **10** bis guide RNA **13** (see Figure 3b). Furthermore, we were restricted in the excess of applied NHS ester, particularly for the branched linker. This led to a situation where an additional urea PAGE purification of the modified guide RNA product was required to separate the final product from unreacted guide RNA, which was time-consuming and largely limited scaling-up and yield. As shown in our recent study, our most advanced guide RNA design, using the branched linkers **8** and **10**, modified with either two BG or two CA self-labeling moieties per linker, clearly improved guide RNA potency, leading to a majorly improved editing performance [19,31,32]. Thus, routinely achieving access to guide RNAs carrying two self-labeling moieties is highly desired. However, the synthesis and purification of such branched linkers, as well as their installment on the guide RNA, is particularly cumbersome and led to comparably low yields compared to the linear linkers. To obtain guide RNAs with two self-labeling moieties from NHS ester **3** or **6**, we purchased guide RNAs **12** and **14,** which carry two amino linkers, a 5′-terminal C12-amino linker and an additional C6-amino-dT building block as the first 5′-terminal nucleotide (see Figure 3a). The latter was easily feasible as our SNAP-ADAR/Halo-ADAR guide RNAs usually contain three nonbinding and thus freely definable nucleotides at their 5′-terminus. To install the respective linker on guide RNA **12** or **14**, respectively, we applied a 40-fold excess (20-fold per amino linker) of the respective NHS ester **3** or **6**. Typically, we obtained full conversion of the guide RNA within one hour of reaction time. Besides the main product, neither unmodified nor mono-modified guide RNA was detected in notable amounts by means of PAGE or analytical HPLC (see Figure 3b, Supplementary Figures S5 and S6). We skipped any urea PAGE modification and applied a simple ethanol precipitation protocol to the guide RNA to remove excess NHS ester and other chemicals, obtaining pure, modified guide RNA carrying two self-labeling moieties ready to be transfected for RNA editing experiments, as outlined in the next paragraph.

### 2.3. Benchmarking Guide RNAs from the Old Versus New Protocol for RNA Base Editing

After successful synthesis of bis-functionalized guide RNAs from NHS ester **3** and **6** following the new, simplified protocol, we aimed to demonstrate their functionality in comparison with guide RNAs generated by the old, labor-intensive protocol from NHS esters **8** and **10**. Importantly, the distance between the two self-labeling moieties and their overall distance from the target adenosine differs slightly between the old protocol (branched linker with mono-amino gRNA) and the new protocol (linear linker with bis-amino gRNA) (Figure 3a). Hence, it was important to compare their editing performance side by side. Specifically, we compared the editing efficiency of two BG-guide RNAs made by the old versus new design on the endogenous target STAT1 (Tyr-701-to-Cys) by applying the SNAP-ADAR1Q tool, and we compared two CA-guide RNAs made by the old versus new design on the endogenous target ACTB (Ile-5-to-Val) by applying the Halo-ADAR1Q tool (Figure 3c,d, Supplementary Figure S8). For this, the respective guide RNAs were transfected either into HeLa-PB-SNAP-ADAR1Q cells [32] or 293 Halo-ADAR1Q Flp-In T-REx cells [19], stably expressing the editing tool from the genome. Notably, for both new protocols, the guide RNAs performed comparably well and elicited editing to a very high efficiency, e.g., 70–80% adenosine-to-inosine conversion at the target side. Together, these results highlight the unconfined functionality of guide RNAs, carrying two self-labeling moieties, which were obtained by reacting NHS esters **3** or **6** with bis-amino gRNAs, like **12** and **14**, following our largely simplified protocol.

## 3. Discussion

Our previous protocols for synthesizing guide RNAs for SNAP- or Halo-tagged effectors suffered from several disadvantages, such as low scalability, chemistry skill requirements, chemistry equipment requirements, and overall labor-intensive protocols with cumbersome purification steps. In this work, we largely simplified guide RNA synthesis by providing protocols for simple liquid phase synthesis of NHS esters of self-labeling moieties, which do not require purification besides precipitation or solvent separation, and which are ready to use for attachment to commercially available amino-modified guide RNAs. Importantly, such NHS esters yielded much better and faster turnover in the attachment reaction to the guide RNA, meaning that modified guide RNAs are ready for use in editing reactions without PAGE purification. Finally, we were able to demonstrate that the complex, branched linkers that have been applied to modify one guide RNA with two self-labeling moieties can be substituted without loss in editing performance by starting from guide RNAs that carry two 5′-terminal amino linkers. In summary, the new protocols largely simplify guide RNA synthesis and would even allow for building ready-to-use kits, and may thus remove a roadblock to broader applications of the approach. Furthermore, the protocol will be helpful in scaling up guide RNA synthesis for future in vivo applications of the SNAP-ADAR tool, e.g., for studying post-translational modifications. This was demonstrated recently over a broad range of target sites [32].

The SNAP-ADAR approach would require the constant expression of an exogenous protein compound. Thus, other approaches are favored for the development of RNA base editing therapeutics [34]. In the past few years, guide RNAs harnessing endogenous ADAR have been developed for that purpose. For example, the CLUSTER [37] and LEAPER [38] approaches represent classes of encoded guide RNAs. Recently, their efficacy was improved by guide RNA circularization and proof of concept for the repair of disease-causing mutations was achieved in vivo [39,40,41]. The recruitment of endogenous ADARs with chemically modified antisense oligonucleotides is another very attractive approach. They are more easily dosed, and their effects are fully reversible, making them safer compared to genetically encoded guide RNAs. Here, the development of the RESTORE platform [42] paved the way and a breakthrough was achieved with the AIMer platform [43] achieving site-directed RNA editing in non-human primates. However, SNAP- and Halo-ADAR display a versatile and easily programmable tool for studying the effect of RNA base editing in cellular models and potentially in vivo in the future.

## 4. Methods

### 4.1. General Chemistry

All chemicals were purchased from standard chemical providers and used without further purification unless stated differently.

TLCs were carried out using silica gel F_254_ foils from MERCK (Darmstadt, Germany) and were visualized either under UV light at 254 nm or were stained with ninhydrin (0.2% ninhydrin (*w*/*v*) with 0.5% acetic acid (*v*/*v*) in ethanol).

Analytical HPLC was performed on a UHPLC SHIMADZU system consisting of a SCL-40 system controller, one DGU-403 and one DGU-405 degassing unit, two LC-40D X3 UHPLC pumps, a CTO-40C column oven, and an SPD-M40 UV/VIS detector. For reaction controls during BG-linker synthesis, buffer A_1_ consisted of H_2_O:TFA 100:0.1, and buffer B_1_ of MeCN:H_2_O:TFA 90:10:0.1 using a linear gradient from 5% B_1_ to 95% B_1_ in 24 min or from 5% B_1_ to 50% B_1_ in 12 min. For the analytical column, a Reprosil Pur Basic C18 from Dr. A Maisch HPLC GmbH, Ammerbuch, Germany was used and prewarmed to 30 °C while the autosampler temperature was set to 15 °C. Analytical HPLC was performed to verify the linker coupling to the guide RNAs was performed as described in detail in the Section 4.5.

NMR spectra were measured on a BRUKER Avance III HDX 400 spectrometer at 400.16 MHz for ^1^H spectra or 100.62 MHz for ^13^C spectra, respectively. Chemical shifts were obtained in ppm and calibrated to the signal of the deuterated solvent.

LC/MS spectra were recorded on a SHIMADZU LCMS-2020 with a kinetex C18 column. Buffer A consisted of H_2_O:HCO_2_H 100:0.1, while buffer B consisted of MeCN:H_2_O:HCO_2_H 80:20:0.1. A linear gradient from 5% B to 95% B in 10 min was applied.

### 4.2. Synthesis of BG-GLA-NHS (**3**)

BG-GLA-NHS (**3**) was synthesized starting from commercially available BG-NH_2_ (Activate Scientific, Prien, Germany, no. AS39187; **1**) in two steps, with minor changes to a published protocol from Park et al. 2018 [36]. In a first step, BG-GLA-OH (**2**) was synthesized by dissolving 1 g (1.0 eq) BG-NH_2_ in 20 mL dry dimethyl acetamide (DMA) by constant stirring and heating to 80 °C for 5 min. After letting the solution cool down to room temperature, DIPEA (1.2 eq) and DMAP (0.5 eq) dissolved in 3.0 mL DMA were added. Subsequently, glutaric anhydride (1.2 eq) dissolved in 4 mL DMA was added slowly. The reaction was stirred for 1 h at room temperature and was monitored by TLC (MeOH:DCM:acetic acid 3:7:0.1) and HPLC. The complete reaction was transferred to 200 mL water, stirred for 15 min at room temperature, and the pH was adjusted to 4–5 using 1 N HCl to precipitate the product BG-GLA-OH (**2**). Note: product **2** is very acid-sensitive and tends to lose guanine if the pH is not well controlled at this stage. The product was pelleted by centrifugation (10 min at 2200× *g*, 4 °C) and the supernatant was discarded. Three wash steps were performed by resuspending the pellet in 150 mL water and subsequent centrifugation (10 min at 2200× *g*, 4 °C). The pellet was resuspended in a small amount of water and dried by lyophilization. The product was stored at −20 °C, protected from moisture. See the Supplementary Materials for the HPLC reaction control and NMR spectra.

Yield: 81%, 1.15 g

TLC: MeOH:DCM:acetic acid 3:7:0.1, R_F_ = 0.7

HPLC: RT_BG-GLA-OH_ = 5.9 min

LC-MS: Expected mass [BG-GLA-OH + H]^+^ = 385.14 *m*/*z*, Found mass [BG-GLA-OH + H]^+^ = 385.0 *m*/*z*

HR-MS: expected mass [BG-GLA-OH + H]^+^ = 385.16188 *m*/*z*, found 385.16181 *m*/*z*

^1^H NMR (DMSO-d_6_, 400 MHz): δ_H_ 8.33 (1H, t, *J* = 5.9 Hz), 7.83 (1H, s), 7.45 (2H, d, *J* = 8.1 Hz), 7.26 (2H, d, *J* = 8.1 Hz), 6.27 (2H, s), 5.45 (2H, s), 4.26 (2H, d, *J* = 5.9 Hz), 2.21 (2H, t, *J* = 7.4 Hz), 2.17 (2H, t, *J* = 7.4 Hz), 1.74 (2H, q, *J* = 7.4 Hz)

^13^C NMR (100.6 MHz, DMSO-d_6_): δ = 174.26, 171.67, 159.66, 159.53, 155.91, 139.56, 138.33, 135.22, 128.55, 127.27, 112.90, 66.56, 41.84, 34.44, 33.19, 20.77

In the second step, 400 mg (1.0 eq) BG-GLA-OH (**2**) was dissolved in 10 mL dry DMA and cooled to 0 °C on ice while stirring. N-hydroxy succinimide (1.4 eq) and EDC*HCl (1.4 eq) were added as solids under dry atmosphere. After 1 h, the ice was removed and the reaction was stirred for 24 h at room temperature. The product **3** was precipitated in pre-cooled water (40 mL) with a pH between 4 and 6. The precipitate was pelleted by centrifugation (10 min at 5000× *g*, 4 °C), the supernatant discarded, and the pellet washed once by resuspending in 40 mL water and subsequent centrifugation. The pellet was resuspended in a small amount of water, lyophilized, and stored at −20 °C protected from moisture. We recommend running the reactions under a dry atmosphere; for example, with a nitrogen balloon. See the Supplementary Materials for the HPLC reaction control and NMR spectra.

Yield: 64%, 332 mg

TLC: MeOH:DCM 1:4, R_F_ = 0.5

HPLC RT_BG-GLA-NHS_ = 7.2 min

LC-MS: Expected mass [BG-GLA-NHS + H]^+^ = 482.17 *m*/*z*, Found mass [BG-GLA-NHS + H]^+^ = 482.2 *m*/*z*

HR-MS: expected mass [BG-GLA-NHS + H]^+^ = 482.17826 *m*/*z*, found 482.17855 *m*/*z*

^1^H NMR (400 MHz, DMSO-d_6_): δ = 12.46 (1H, s), 8.39 (1H, t, *J* = 5.9 Hz), 7.84 (1H, s), 7.45 (2H, d, *J* = 8.1 Hz), 7.26 (2H, d, *J* = 8.1 Hz), 6.29 (2H, s), 5.46 (2H, s), 4.27 (2H, d, *J* = 5.8 Hz), 2.81 (4H, s), 2.70 (2H, t, 7.4 Hz), 2.27 (2H, t, 7.4 Hz), 1.87 (2H, q, 7.4 Hz).

^13^C NMR (100.6 MHz, DMSO-d_6_): δ = 171.17, 170.27, 168.79, 159.63, 159.50, 156.06, 139.47, 138.41, 135.22, 128.56, 127.29, 112.57, 66.58, 41.89, 33.63, 29.69, 25.47, 20.41

### 4.3. Synthesis of CA-GLA-NHS (**6**)

Synthesis of CA-GLA-NHS (**6**) was performed analogously to that of BG-GLA-NHS (**3**) with minor changes. First, 1.0 g (1.0 eq) CA-NH_2_ (BLD pharm, Shanghai, China, no. BD01138570; **4**) was dissolved in 40 mL dry DCM, glutaric anhydride (1.2 eq) was added as a solid, and subsequently, DIPEA (2 eq) and DMAP (0.5 eq) dissolved in 10 mL DCM were added to the reaction. The reaction was stirred for 1 h at room temperature. A total of 50 mL aqueous acetic acid (1%) was added, and the mixture was stirred for 10 min. Organic and aqueous phases were separated by a separating funnel, with the product **5** in the organic phase. The DCM was removed by rotary evaporation and the CA-GLA-OH (**5**) was further dried by lyophilization and stored at −20 °C, protected from moisture. See the Supplementary Materials for the HPLC reaction control and NMR spectra.

Yield: 62%, 468 mg

LC-MS: Expected mass [CA-GLA-OH + H]^+^ = 337.84 *m*/*z*, Found mass [CA-GLA-OH + H]^+^ = 337.95 *m*/*z*

^1^H NMR: (DMSO, 400 MHz): δ_H_ 7.84 (1H, t, *J* = 5.5), 3.62 (2H, t, *J* = 6.6), 3.51–3.45 (4H, m), 3.40–3.35 (4H, m), 3.20–3.15 (2H, q, *J* = 5.8), 2.19 (2H, t; *J* = 7.4), 2.09 (2H, t, *J* = 7.5), 1.74–1.66 (4H, m), 1.49 (4H, quint.), 1.42–1.26 (4H, m)

For activation, 125 mg of CA-GLA-OH (**5**) was dissolved in 10 mL dry DCM and cooled down on ice to 0 °C. Then, NHS (1.2 eq) and EDC-HCl (1.2 eq) dissolved in 10 mL DCM were added, and the reaction was stirred for 24 h while reaching room temperature. For workup, 20 mL 1 M sodium bicarbonate was added and the organic phase containing product **6** was separated using a separating funnel. The organic phase was washed with 20 mL 1 M HCl and brine solution in the separating funnel. The DCM was removed by rotary evaporation and subsequently high vacuum was applied to remove remaining water and the dried product was stored at −20 °C, protected from moisture. We recommend running the reactions under a dry atmosphere; for example, with a nitrogen balloon. See the Supplementary Materials for the HPLC reaction control and NMR spectra.

Yield: 76%, 122.5 mg

LC-MS: Expected mass [CA-GLA-NHS + H]^+^ = 435.05 *m*/*z*, Found mass [CA-GLA-NHS + H]^+^ = 434.91 *m*/*z*

HR-MS: expected mass [CA-GLA-NHS + Na]^+^ = 457.17120 *m*/*z*, found [CA-GLA-NHS + Na]^+^ = 457.17142 *m*/*z*

^1^H NMR (DMSO, 400 MHz): δ_H_ 7.92 (1H, t, *J* = 5.5), 3.62 (2H, t, *J* = 6.6), 3.51–3.45 (4H, m), 3.42–3.33 (4H, m), 3.21–3.17 (2H, q, *J* = 5.8), 2.81 (4H, s), 2.68 (4H, t, *J* = 7.5), 2.19 (2H, t, *J* = 7.4), 1.82 (2H, quint., *J* = 7.3), 1.70 (2H, quint., *J* = 7.8), 1.49 (4H, quint., *J* = 7.3), 1.42–1.24 (4H, m)

^13^C NMR (100.6 MHz, DMSO-d_6_): δ = 171.19, 170.21, 168.74, 70.17, 69.55, 69.42, 69.06, 45.36, 38.47, 33.63, 32.00, 29.62, 29.04, 26.11, 25.44, 24.91, 20.40

### 4.4. Protocol for Attachment of Self-Labeling Moieties **3** (BG) and **6** (CA) to Guide RNAs

All amino guide RNAs were purchased from Biospring (Frankfurt am Main, Germany) or Eurogentech (Seraing, Belgium) either HPLC-purified or desalted. The sequences, full modification patterns, and extinction coefficients at 260 nm for all guide RNAs can be found in Table 1. Amino guide RNAs are typically 22–25 nt long, stabilized by 2′-O-methylation outside the central base triplet, carrying few locked nucleic acid building blocks and terminal phosphorothioate linkages [19,32]. Additionally, one or two amino linkers are included for the attachment of one or two self-labeling moieties. Guide RNAs carrying the previously reported self-labeling moieties and linker designs were synthesized and purified as described before [19,35].

For the attachment of the respective self-labeling moiety, BG or CA, to the guide RNAs, a 100 mM stock solution of the respective NHS ester (**3** or **6**) was prepared in DMSO. Typically, 5 nmol of the guide RNA was diluted in 40 µL coupling buffer (1:20 0.2 M NaHCO_3_ buffer pH 9 in PBS) plus 8 µL DMSO. Then, 2 µL of the linker stock solution was added (40 eq, 80 nmol) and the reaction was incubated at 37 °C (900 rpm) for 1 h. The reaction can be monitored by analytical HPLC, as described below. To finish the reaction, the mixture was frozen and lyophilized. The mixture was dissolved in 150 µL nuclease free-water, and precipitated by adding 0.1 volume of 3 M sodium acetate and a threefold excess of cold isopropanol, followed by incubation overnight at −20 °C. The precipitated guide RNA was pelleted by centrifugation (18,000× *g*, −4 °C, 1 h); the supernatant was removed carefully and the pellet washed twice by adding 100 µL ice-cold 70% isopropanol and subsequent centrifugation (18,000× *g*, −4 °C, 45 min). After removing the isopropanol, the pellet was briefly dried at 70 °C (~3 min) and resuspended in 20 µL nuclease-free water. The concentration and amount of the guide RNA was determined by UV (nanodrop). We typically obtained yields between 70 and 85% after precipitation. Purity can also be confirmed by analytical HPLC (see below). Typically, we saw full conversion of all amino linkers and the complete removal of any traces of **3** and **6**. Guide RNAs were used without any further purification in subsequent editing reactions.

### 4.5. Analytical HPLC of Guide RNA Reactions

Analytical HPLC was performed on an UHPLC SHIMADZU system consisting of a SCL-40 system controller, one DGU-403 and one DGU-405 degassing unit, two LC-40D X3 UHPLC pumps, a CTO-40C column oven, and an SPD-M40 UV/VIS detector. Reaction controls for the coupling of the NHs esters **3** or **6** to the amino guide RNAs were performed on a Reprosil Pur Basic C18 column from Dr. A Maisch HPLC GmbH using different buffers. Specifically, a TEAA buffer was prepared by adding glacial acid dropwise to an aqueous solution of triethylamine (1 M) in a 1:1 molar ratio while stirring on ice. The pH was adjusted to 7.2 by titrating acetic acid. The solution was then diluted to 0.1 M with nanopure water. To prepare buffer A_2_ (TEAA buffer:MeCN 95:5), acetonitrile was added to the TEAA solution to a final concentration of 5% (*v*/*v*) and sterile filtered (0.22 µm polyethersulfone filters). Buffer B_2_ consisted of MeCN:H_2_O 90:10. Here, linear gradients from 5% B_2_ to either 95% B_2_ (in 24 min), 50% B_2_ (in 12 min), or 38.75% B_2_ (in 9 min) were applied to the column, which was prewarmed to 45 °C. The autosampler’s temperature was set to 4 °C. All spectra were analyzed with SHIMADZU Lab Solutions (Nakagyo-ku, Japan).

### 4.6. Analytical Urea PAGE of Guide RNA Reactions

To assess the purity and turnover of the guide RNAs with the self-labeling moieties, analytical urea PAGE was performed. Specifically, samples were mixed with 0.5 volumes RNA loading dye (50% urea in 1x TBE containing bromophenol blue (~0.025%) and Xylene cyanol (~0.025%) and run on a 20% acrylamide (acrylamide:bisacrylamide 19:1) 6.66 M urea PAGE [44]. The PAGE was run for ~300 min at 1.4 kV and imaged using fluorescent TLC Silica gel 60 F_254_ plates (Merck) under short wave (254 nm) UV light. 

### 4.7. Cell Culture, RNA Editing of Endogenous Targets

In general, cells were cultivated in Dulbecco’s modified Eagle’s medium (DMEM, Life Technologies, Waltham, MA, USA) supplemented with 10% fetal bovine serum (FBS, Life Technologies) at 37 °C, 5% CO_2_ in a water-saturated humid atmosphere. The previously published HeLa-PB-SA1Q cell line [32] (ACC 57, DSMZ, Braunschweig, Germany) was used for STAT1 and the reported Flp-In HAQ1 cell line [19] (cat. no. R78007 ThermoFisher Scientific, Waltham, MA, USA) for ACTB editing experiments, respectively.

Editing of STAT1 in HeLa-PB-SA1Q cells was performed as previously reported [32]. First, 4 × 10^5^ cells were seeded in DMEM/FBS/1 µg/mL doxycycline. After 24 h, 5 × 10^4^ cells were reverse transfected with 2 pmol guide RNA with 0.75 µL Lipofectamine 2000 following the manufacturer’s instructions. Doxycycline concentration was kept at 1 µg/mL and at 24 h post-transfection, cells were harvested.

Editing of ACTB in Flp-In HAQ1 cells was performed as reported previously [19]. Briefly, 4 × 10^5^ cells were seeded in DMEM/FBS/B/H/10 ng/mL doxycycline. After 24 h, 8 × 10^4^ cells were reverse transfected with 50 nM guide RNA (5 pmol) with 0.5 µL Lipofectamine 2000 following the manufacturer’s instructions. Doxycycline concentration was kept at 10 ng/mL and at 24 h post-transfection, cells were harvested.

RNA isolation was performed using the Monarch RNA cleanup kit from NEB (Ipswich, MA, USA) and subsequent DNase I digestion. RT-PCR was performed using the OneStep OneTaq RT-PCR kit from NEB following the instructions, for which the primer sequences can be found in Supplementary Table S1. PCR products were purified by agarose gel extraction and subsequently analyzed with Sanger sequencing (Microsynth, Balgach, Switzerland). A-to-I editing yield was calculated by dividing the peak height for guanosine by the sum of the peak heights for guanosine and adenosine.

## Figures and Tables

**Table 1:** Sequences, modification patterns, and extinction coefficients (ε_260 nm_) of all guide RNAs. Italic text indicates 2′OMe-rNT, underlined indicates ribonucleotides, * indicates phosphorothioate linkage, {N} indicates LNA base. Y indicates internal C6-Amino-dT (the structure is displayed in Supplementary Figure S6).

**Guide RNA**		**Target**	**Sequence**	**ε_260nm_/mM^−1^cm^−1^**
Mono-amino STAT1	**11**	STAT1 Y701C	5’-C6-amino-A*G*U{G}*U*{C}*UUGAU*ACAUCCAGUU*C*{C}*U*{T}	255
Bis-amino STAT1	**12**	STAT1 Y701C	5’-C12-amino-*Y*G*U{G}*U*{C}*UUGAU*ACAUCCAGUU*C*{C}*U*{T}	250
Mono-amino ACTB	**13**	ACTB I5V	5’-C6-amino-C*U*CCGCGGCG ACA UCAUC*A*U*C*C	200
Bis-amino ACTB	**14**	ACTB I5V	5’-C12-amino-*Y*C*U*CCGCGGCG ACA UCAUC*A*U*C*C	215

## Data Availability

The data supporting the findings of this study are available within the Supplementary Materials files and/or from the corresponding author upon request.

## Supplementary Materials

The following supporting information can be downloaded at: https://www.mdpi.com/article/10.3390/molecules30051049/s1, Figure S1. Chemical structures of previously used linkers. Figure S2. Reaction and purification controls during BG-GLA-OH synthesis. Figure S3. Reaction and purification controls during BG-GLA-NHS synthesis. Figure S4. HPLC control of the in-situ pre-activation of the old BG-OH v1 (**7**). Figure S5. HPLC reaction controls for guide RNA synthesis with old versus new BG linkers. Figure S6. HPLC reaction controls for guide RNA synthesis with the old versus new CA linker. Figure S7. Structure of C6-Amino-dT with PS linkages. Figure S8. Exemplary sequencing traces for Figure 3c. Table S1. Primer sequences and annealing temperature for amplifying target.
