# Supplementary material for: A Simplified Guide RNA Synthesis Protocol for SNAP- and Halo-Tag-Based RNA Editing Tools
Source: Molecules. 2025 Feb 26;30(5):1049. doi: 10.3390/molecules30051049 (PMC11901542; doi:10.3390/molecules30051049)

Supporting Information

A simplified guide RNA synthesis protocol for SNAP- and Halo-tag-based RNA editing tools

Daniel Tobias Hofacker[1], Sebastian Kalkuhl[1], Jana Franziska Schmid[1], Shubhangi Singh[1] and Thorsten Stafforst[1,2]*

[1]Interfaculty Institute of Biochemistry, University of Tübingen (Germany)

[2]Gene and RNA Therapy Center (GRTC), Faculty of Medicine, University Tübingen (Germany)

*correspondence to thorsten.stafforst@uni-tuebingen.de

Contents

Previously used linkers	3
Reaction controls for BG-GLA-OH synthesis	4
Reaction controls for BG-GLA-NHS synthesis	5
Pre-activation of BGv1	6
Generation of guide RNAs	7
Structure of Amino-dT-C6	9
Exemplary Sequencing traces for RNA editing experiments	9
Constructs for stable cell lines	10
Supporting literature	10
Appendix	11
^{1}H NMR spectrum for BG-GLA-OH	11
^{13}C NMR spectrum for BG-GLA-OH	11
^{1}H-^{1}H COSY for BG-GLA-OH	12
^{1}H-13C HSQC for BG-GLA-OH	12
^{1}H NMR spectrum for BG-GLA-NHS	13
^{13}C NMR spectrum for BG-GLA-NHS	13
^{1}H-^{1}H COSY for BG-GLA-NHS	14
^{1}H-13C HSQC for BG-GLA-NHS	14
^{1}H-13C HMBC for BG-GLA-NHS	15
^{1}H NMR spectrum for CA-GLA-OH	15
^{1}H NMR spectrum for CA-GLA-NHS	16
^{13}C NMR spectrum for CA-GLA-NHS	16
^{1}H-^{13}C HSQC NMR spectrum for CA-GLA-NHS	17
Uncropped PAGE images for Figure 3b	18

a

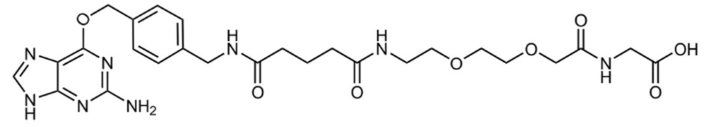

BG-OH v1 (7)

b

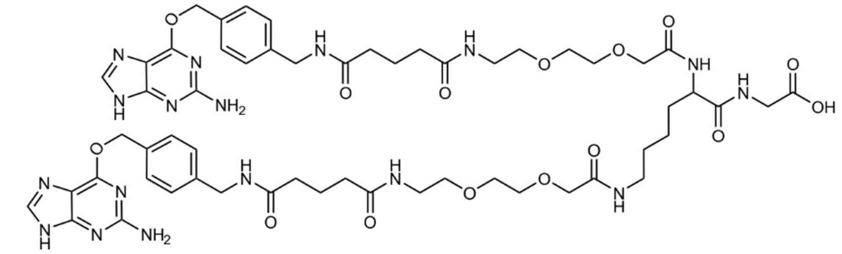

BisBG-OH v1 (8)

c

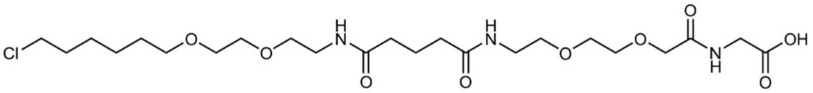

CA-OH v1 (9)

d

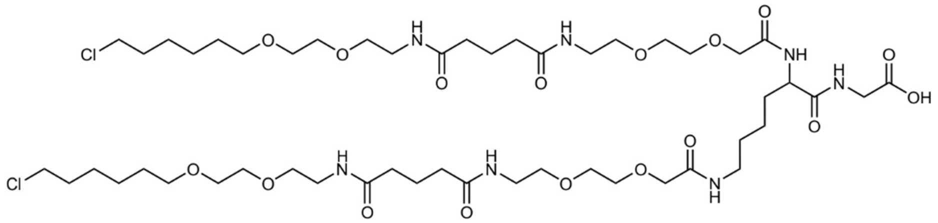

BisCA-OH v1 (10)

Supplementary Fig. S1. Chemical structures of previously used linkers. a) Structure of BG-OH_v1 **(7).** Literature-known BG-OH_v1 was synthesized from commercially available *O*^{6}-(4-aminomethyl-benzyl)guanine (BG-NH_2) according to previously published protocols[1]. **b)** Structure of BisBG-OH_v1 **(8).** Literature-known BisBG-OH_v1 was synthesized from commercially available *O*^{6}-(4-aminomethyl-benzyl)guanine (BG-NH_2) according to previously published protocols[2]. **c)** Structure of CA-OH_v1 **(9).** Literature-known CA-OH_v1 was synthesized from commercially available 2-(2-aminoethoxy)ethanol according to previously published protocols[2]. **d)** Structure of BisCA-OH_v1 **(10).** Literature-known BisCA-OH_v1 was synthesized from commercially available 2-(2-aminoethoxy)ethanol according to previously published protocols[2]. Before coupling to a guide RNA, all linkers require pre-activation as an NHS ester.

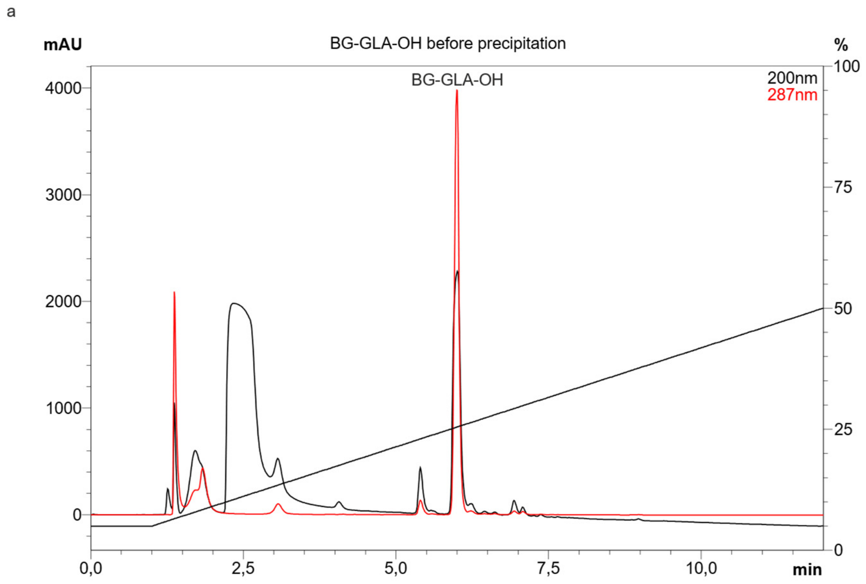

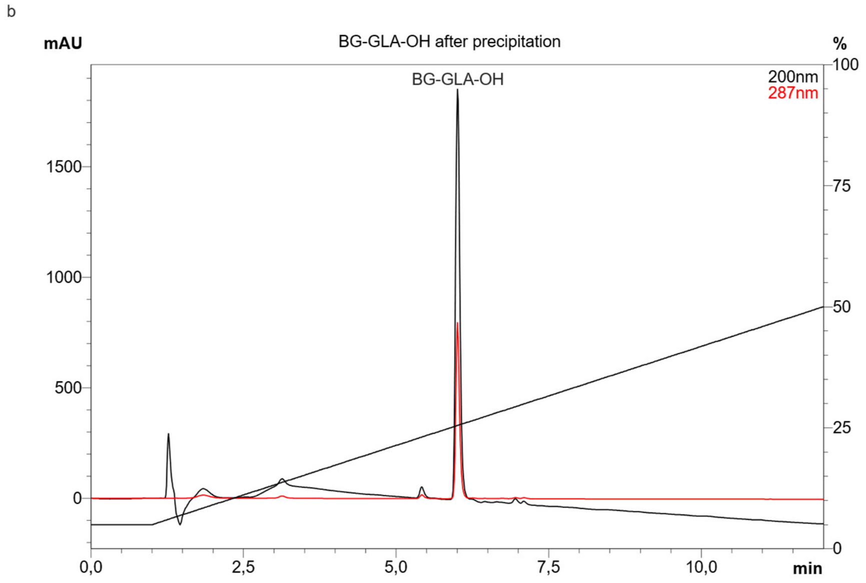

Supplementary Fig. S2. Reaction and purification controls during BG-GLA-OH synthesis. a) HPLC trace of crude BG-GLA-OH **(2).** t_R (BG-GLA-OH) = 5.9 min **b)** HPLC trace of precipitation-purified BG-GLA-OH **(2).** t_R (BG-GLA-OH) = 5.9 min

a

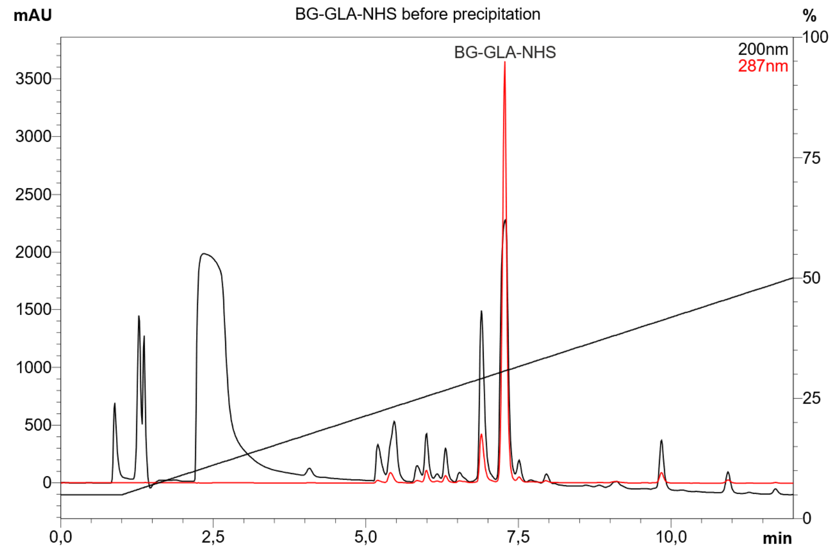

b

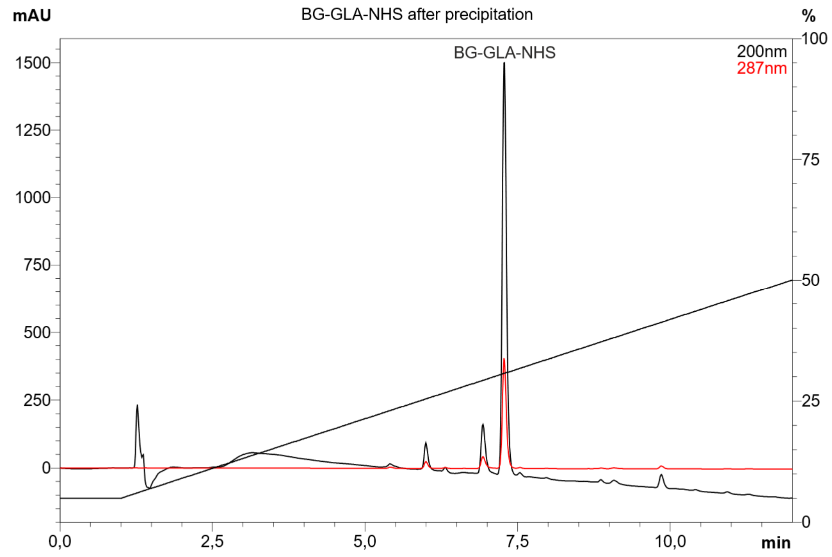

Supplementary Fig. S3. Reaction and purification controls during BG-GLA-NHS synthesis. a) HPLC trace of crude BG-GLA-NHS **(3).** t_R (BG-GLA-NHS) = 7.2 min **b)** HPLC trace of precipitation-purified BG-GLA-NHS **(2).** t_R (BG-GLA-NHS) = 7.2 min

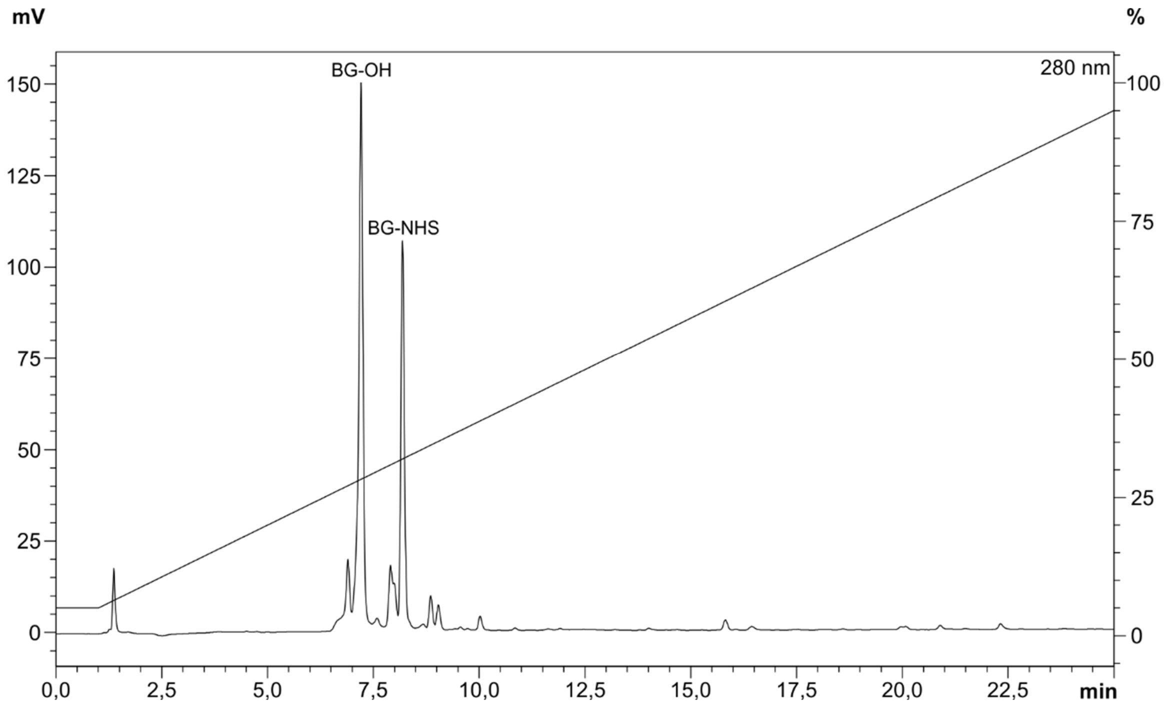

Supplementary Fig. S4. HPLC control of the in-situ pre-activation of the old BG-OH v1 (7). The reaction for pre-activation of BG-OH v1 **(7)** was carried out as published previously[1] and monitored using a SHIMADZU HPLC consisting of a SCL-10A VP system controller, two LC-20AT prominence liquid chromatographs for buffers A and B, and an SPD-20AV prominence UV/VIS detector. Buffer A consisted of H2O:TFA, 100:0.1, buffer B of MeCN:H2O:TFA, 90:10:0.1. For analytic measurements, a linear gradient from 5 % B to 95 % B in 25 min was applied. For the analytical column, an EC 125/4 nucleodur 100-5 C18 ec column by MACHEREY-NAGEL was used. Typically, a turnover of less than 50 % was observed. BG-NHS was used without further purification. t_R(BG-OH) = 7.2 min, t_R(BG-NHS) = 8.25 min

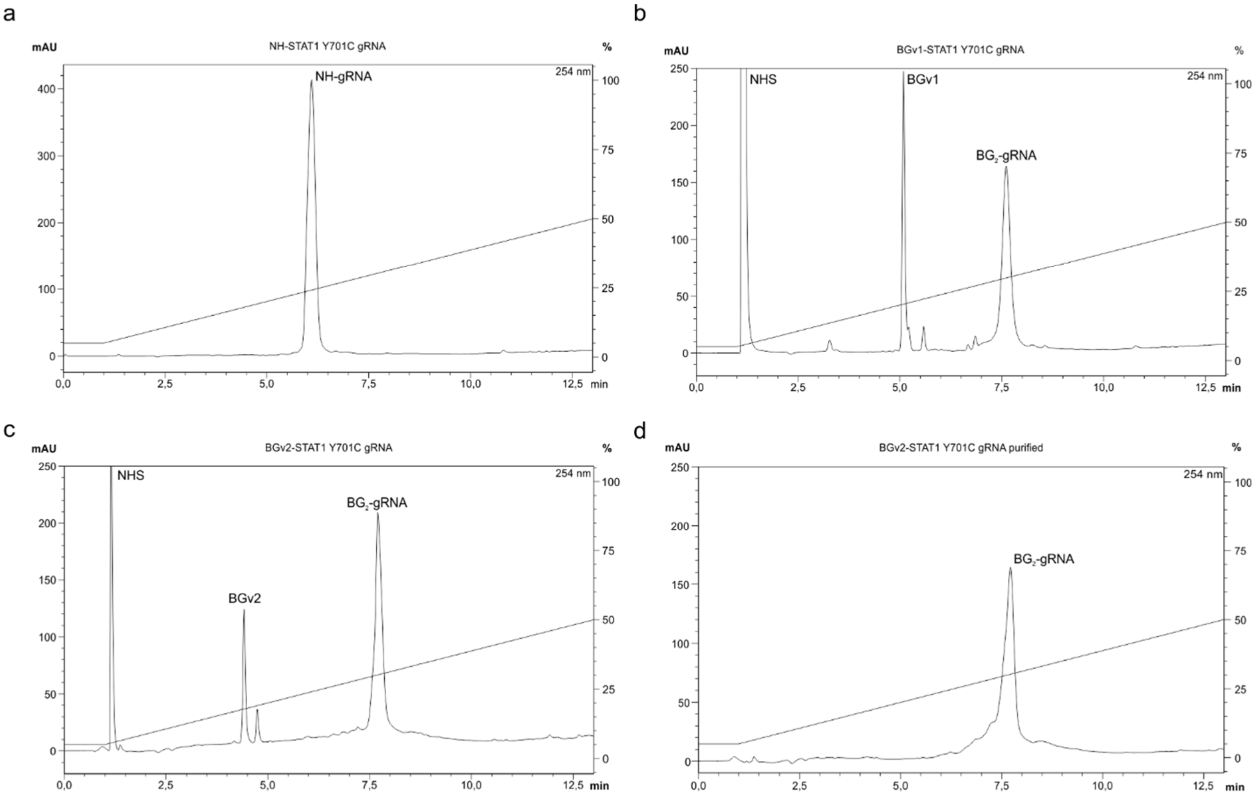

Supplementary Fig. S5. HPLC reaction controls for guide RNA synthesis with old versus new BG linkers. For BG-coupling to the STAT1 Y701 guide RNA, 40 µg guide RNA **12** was used per reaction with a 40-fold excess of linker. In each run, approximately 2.5 µg guide RNA was loaded onto HPLC. The absorption at 254 nm is shown and a gradient from 5 % buffer B to 50 % buffer B was used as described in the Methods section. **a)** HPLC of unmodified NH_2-STAT1 Y701C guide RNA. t_R = 6.1 min. **b)** HPLC of unpurified STAT1 Y701C guide RNA modified with BGv1 (**7**). t_R = 7.55 min. Other peaks belong to released NHS and uncoupled BGv1 (**7**). Further urea PAGE was required prior to use in editing applications. **c)** HPLC of unpurified STAT1 Y701C guide RNA modified with BGv2 (**3**). t_R = 7.6 min. Other peaks belong to released NHS and uncoupled BGv2 (**3**). **d)** HPLC of STAT1 Y701C gRNA modified with BGv2 (**3**) after isopropanol precipitation to remove the remaining starting material BGv2 (**3**) and NHS that is released during the reaction. t_R = 7.6 min. The guide RNA was used for subsequent RNA editing applications without further purification.

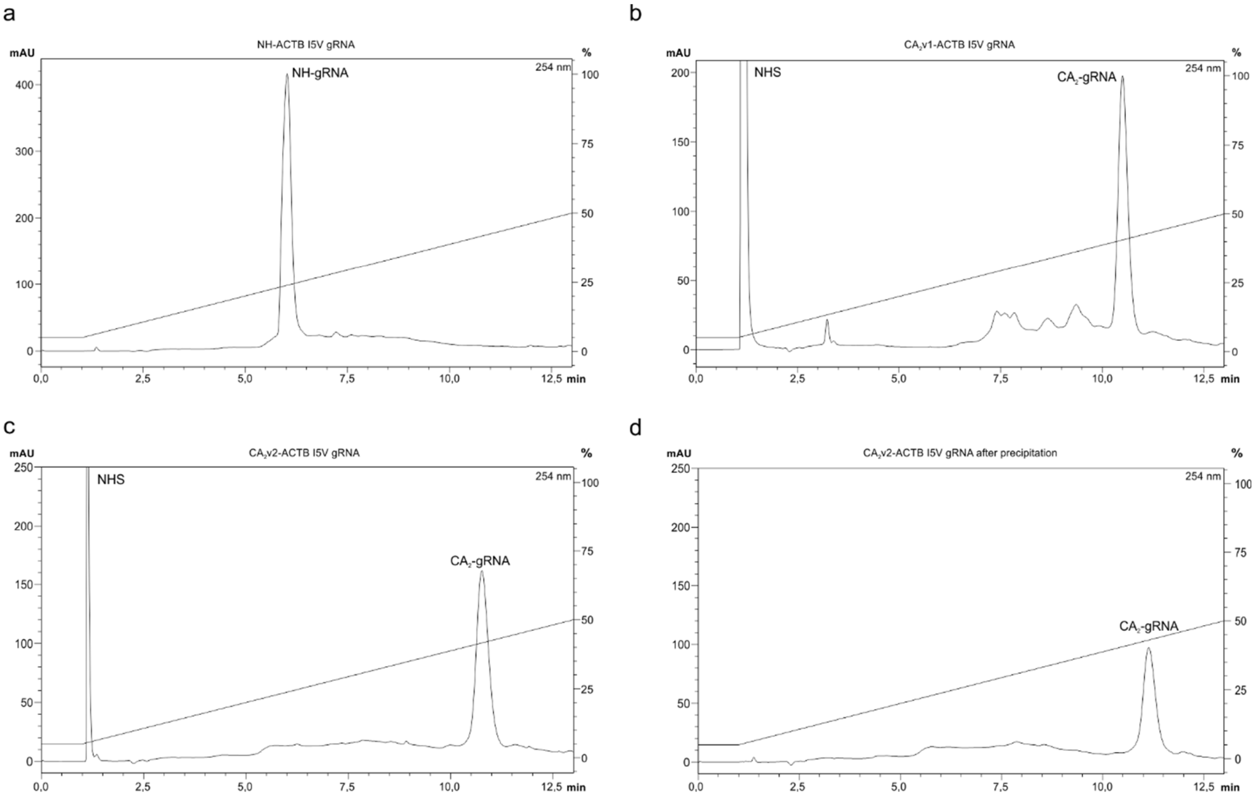

Supplementary Fig. S6. HPLC reaction controls for guide RNA synthesis with the old versus new CA linker. For CA-coupling to the ACTB I5V guide RNA, 40 µg guide RNA **14** was used per reaction with a 40-fold excess of linker. In each run, approximately 2.5 µg guide RNA was loaded onto HPLC. The absorption at 254 nm is shown and a gradient from 5 % buffer B to 50 % buffer B was used as described in the Methods section. **a)** HPLC of unmodified NH_2-ACTB I5V gRNA. t_R = 6.0 min. **b)** HPLC of unpurified ACTB I5V gRNA modified with CAv1 (**9**). t_R = 10.5 min. The Other peak belongs to released NHS. **c)** HPLC of unpurified ACTB I5V gRNA modified with CAv2 (**6**). t_R = 10.75 min. The Other peak belongs to released NHS. **d)** HPLC of purified ACTB I5V gRNA modified with CAv2 (**6**) after isopropanol precipitation to remove the remaining starting material CAv2 (**6**) and NHS that is released during the reaction. t_R = 10.75 min.

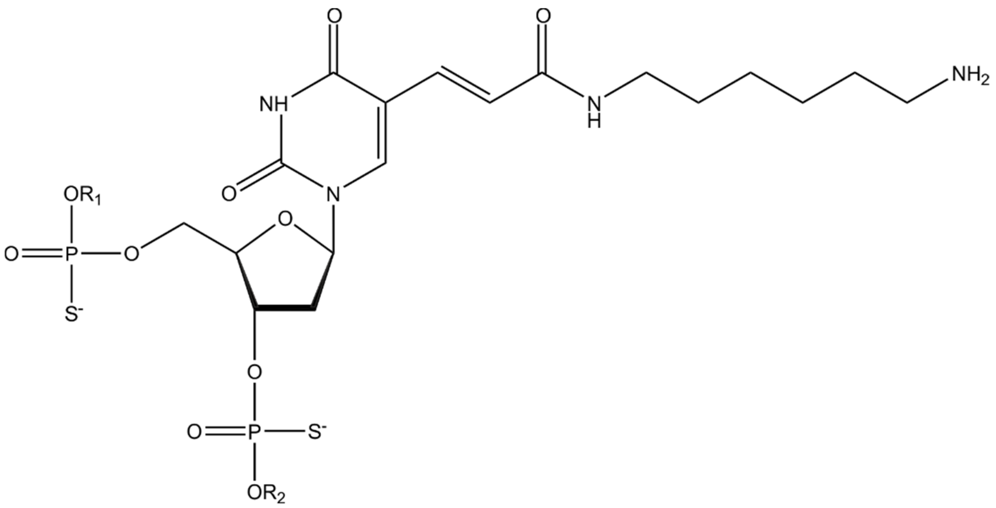

Supplementary Fig. S7. Structure of C6-Amino-dT with PS linkages. The amino group was later modified by either the BG- or CA-linker.

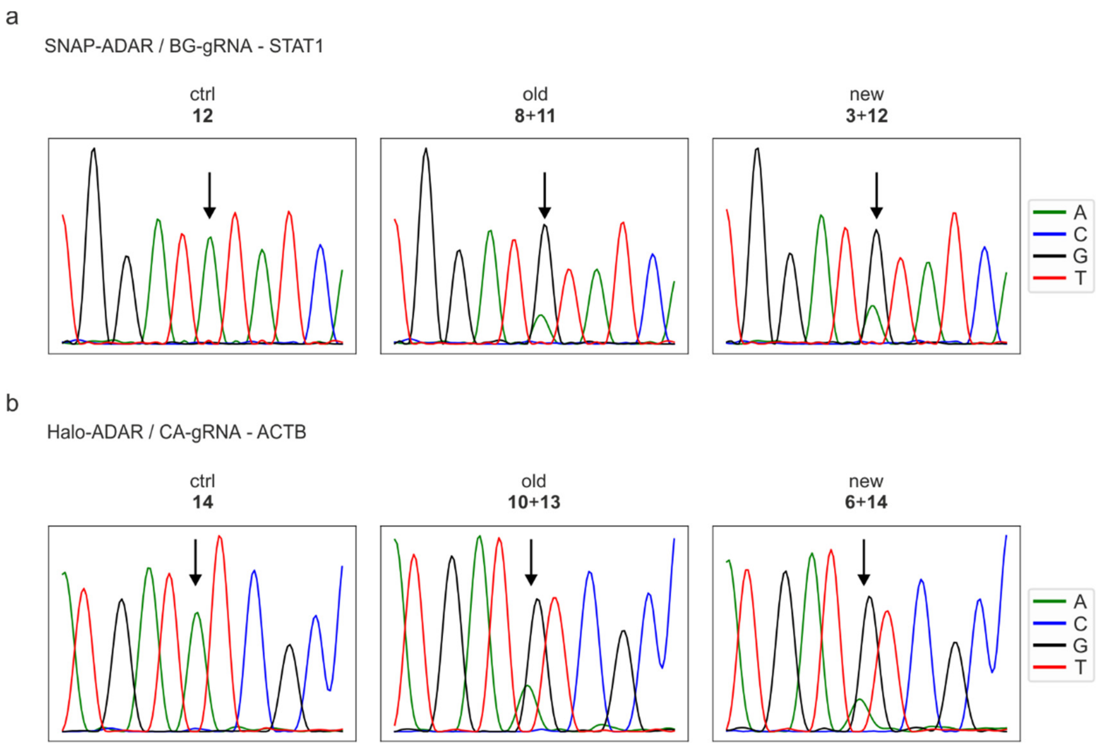

Supplementary Fig. S8. Exemplary sequencing traces for Figure 3c. a) STAT1 Sequencing traces for editing with the SNAP-ADAR system; shown is one representative trace for the negative control (left panel), the old guide RNA design (central panel), and the new guide RNA design (right panel). b) ACTB Sequencing traces for editing with the Halo-ADAR system; shown is one representative trace for the negative control (left panel), the old guide RNA design (central panel), and the new guide RNA design (right panel). The editing experiment was performed as described for Figure 3c.

Supplementary Table S1. Primer sequences and annealing temperature for amplifying target.

Target	Forward primer	Reverse primer	Sense oligo	Temp.	Sequence primer
STAT1 Y701C	gcttcatcagcaaggag cgagagcg	cttcagacacagaaatcaa ctcagtc	aaggaactggatctat caagacacc	55°C	ggctgctgagaatattc ctgagaatc
ACTB I5V	gagcacagagcctcgcc tt	gatgcctctcttgctctgg	-	62°C	gagcacagagcctcgc ctt

Constructs for stable cell lines

The sequences for creating all stable cell lines used in this study can either be found in Stroppel et al. 2021 (Flp-In 293 HA1Q)[2] or Kiran Kumar et al. 2024 (HeLa PB SA1Q)[3].

Supporting literature

1 Vogel, P. *et al.* Efficient and precise editing of endogenous transcripts with SNAP-tagged ADARs. *Nat Methods* **15**, 535-538 (2018). https://doi.org/10.1038/s41592-018-0017-z

2 Stroppel, A. S. *et al.* Harnessing self-labeling enzymes for selective and concurrent A-to-I and C-to-U RNA base editing. *Nucleic Acids Res* **49**, e95 (2021). https://doi.org/10.1093/nar/gkab541

3 Kiran Kumar, K. D. *et al.* An improved SNAP-ADAR tool enables efficient RNA base editing to interfere with post-translational protein modification. *Nat Commun* **15**, 6615 (2024). https://doi.org/10.1038/s41467-024-50395-w

Appendix

^{1}H NMR BG-GLA-OH (2)

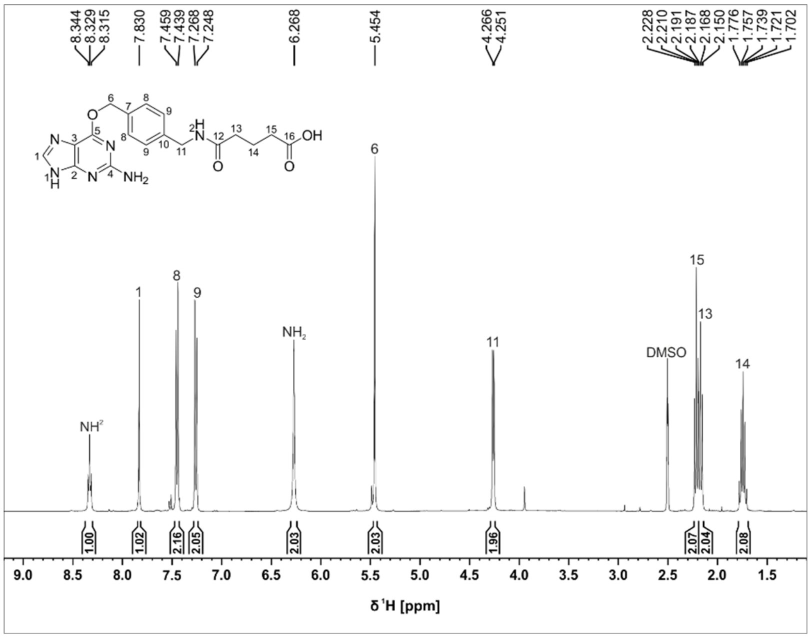

^{13}C NMR BG-GLA-OH (2)

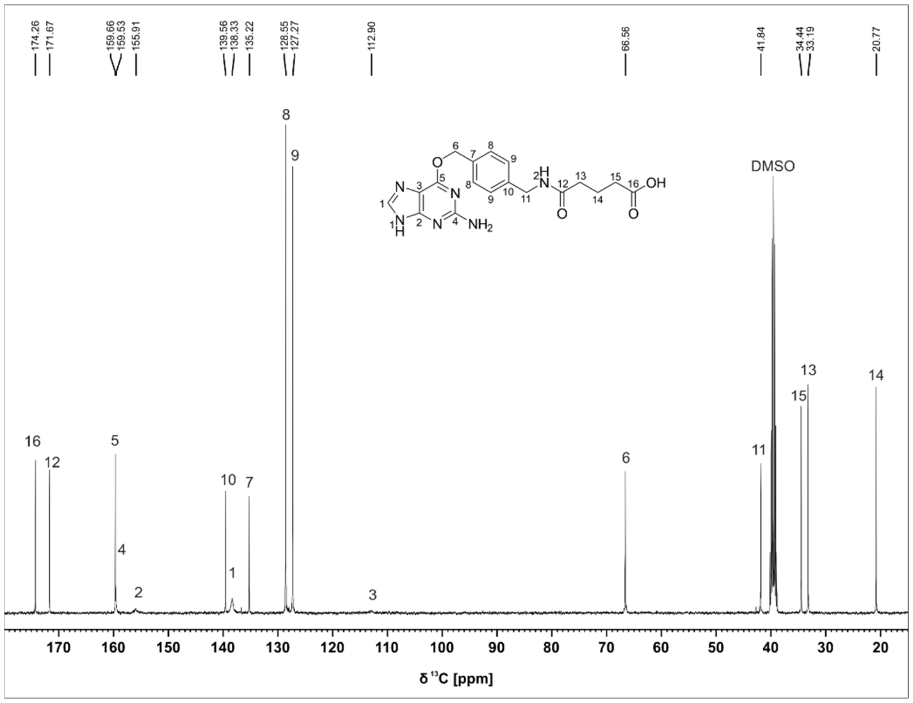

^{1}H-^{1}H COSY BG-GLA-OH (2)

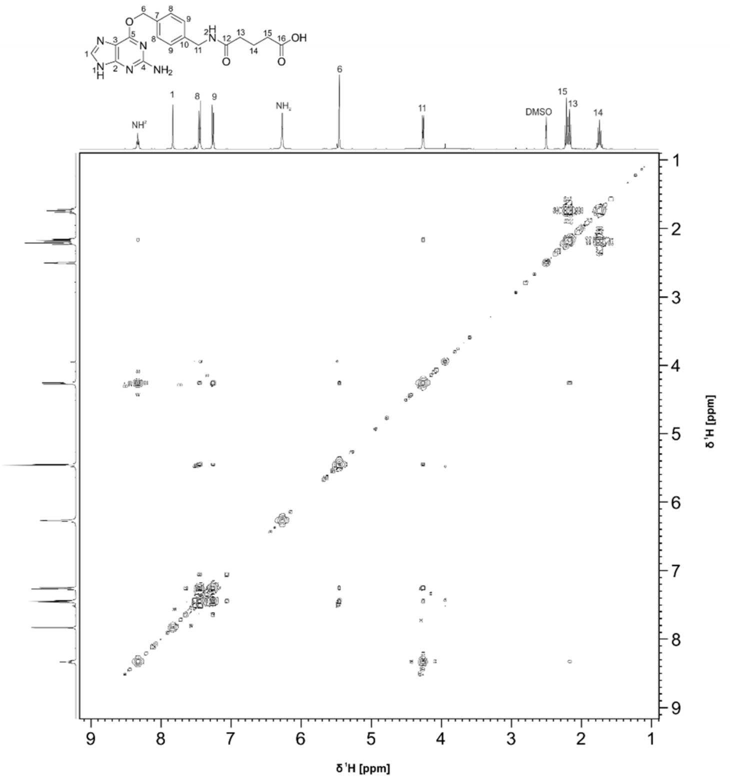

^{1}H-^{13}C HSQC BG-GLA-OH (2)

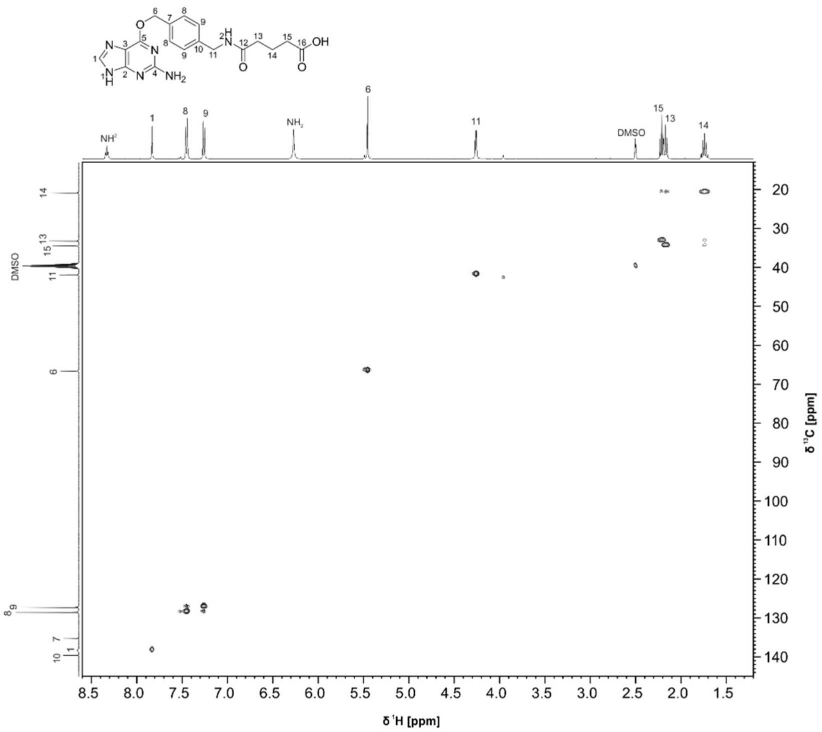

^{1}H NMR BG-GLA-NHS (3)

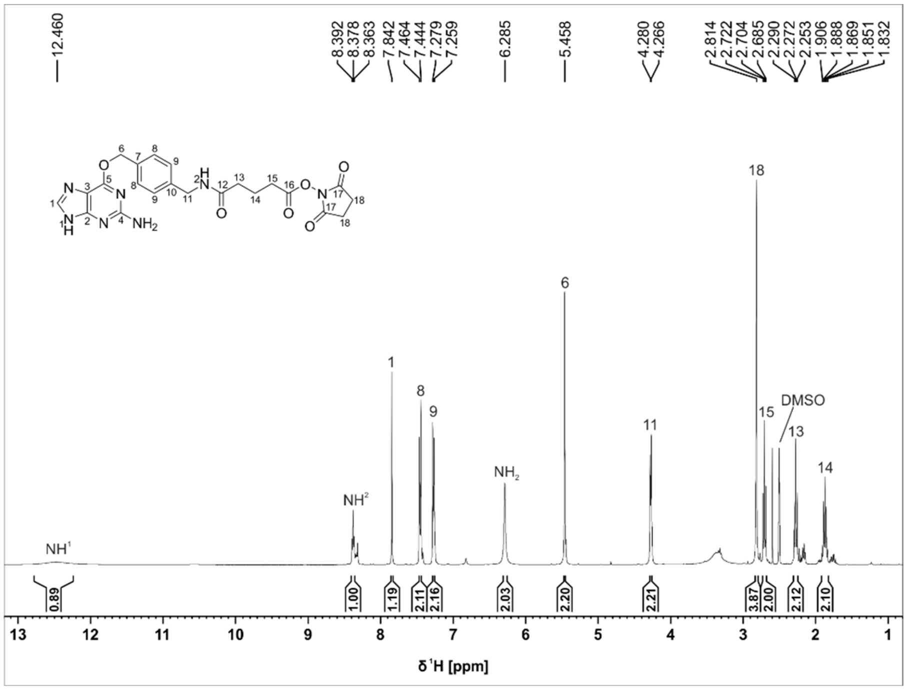

^{13}C NMR BG-GLA-NHS (3)

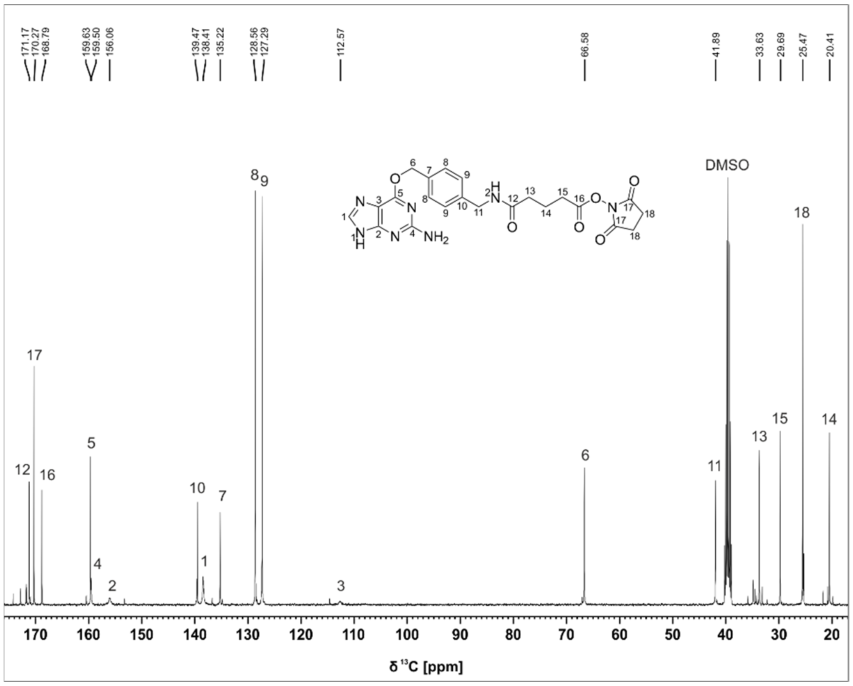

^{1}H-^{1}H COSY BG-GLA-NHS (3)

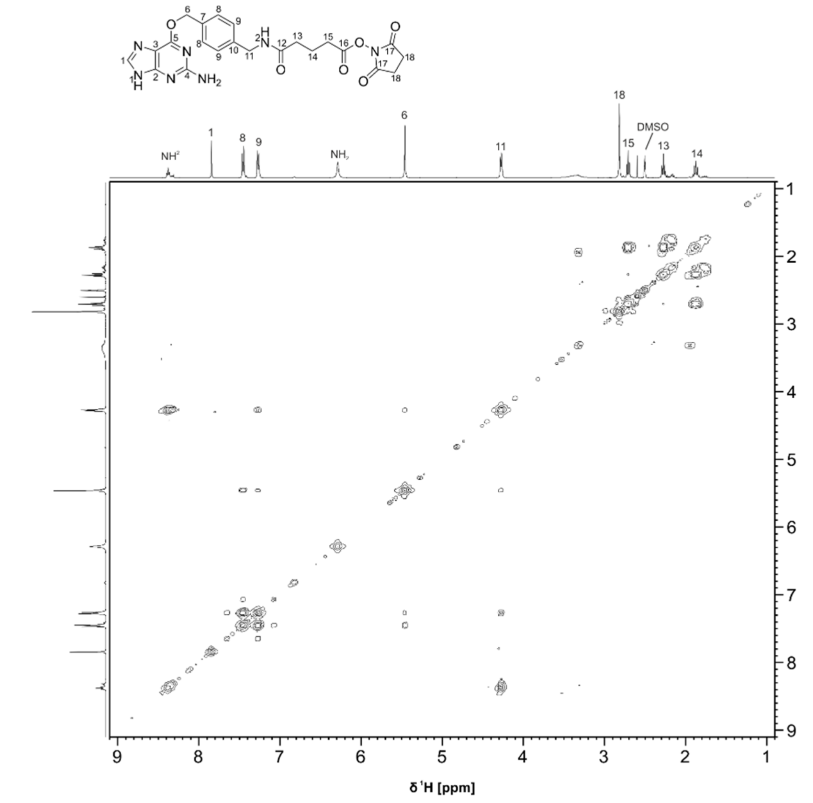

^{1}H-^{13}C HSQC BG-GLA-NHS (3)

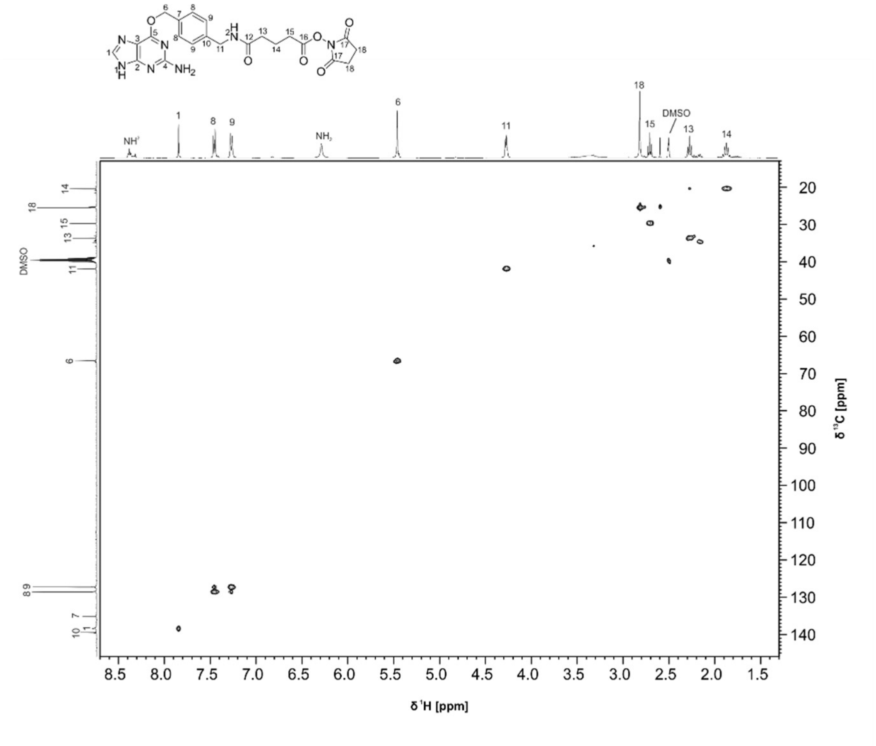

^{1}H-^{13}C HMBC BG-GLA-NHS (3)

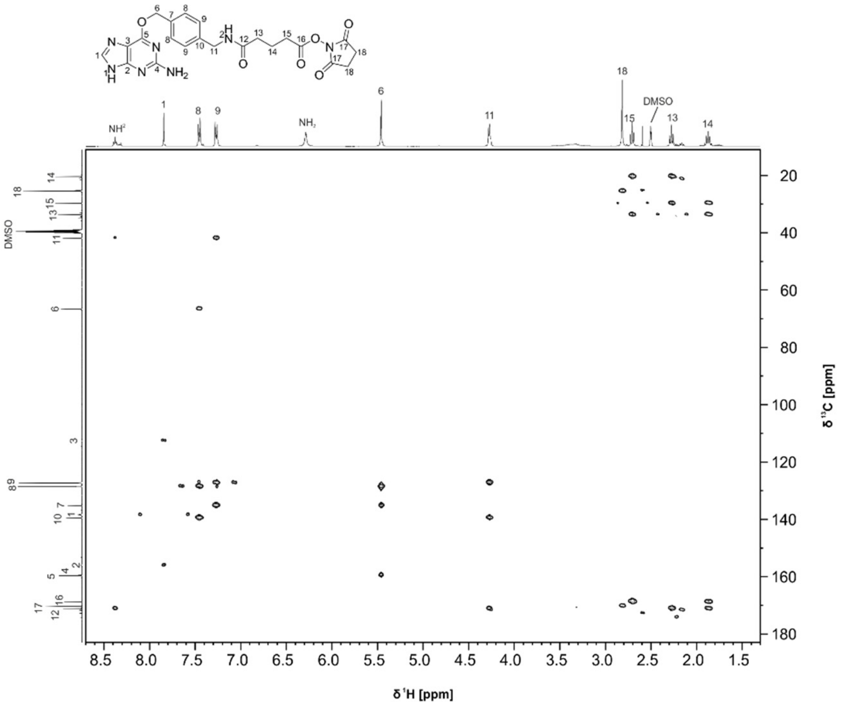

^{1}H NMR CA-GLA-OH (5)

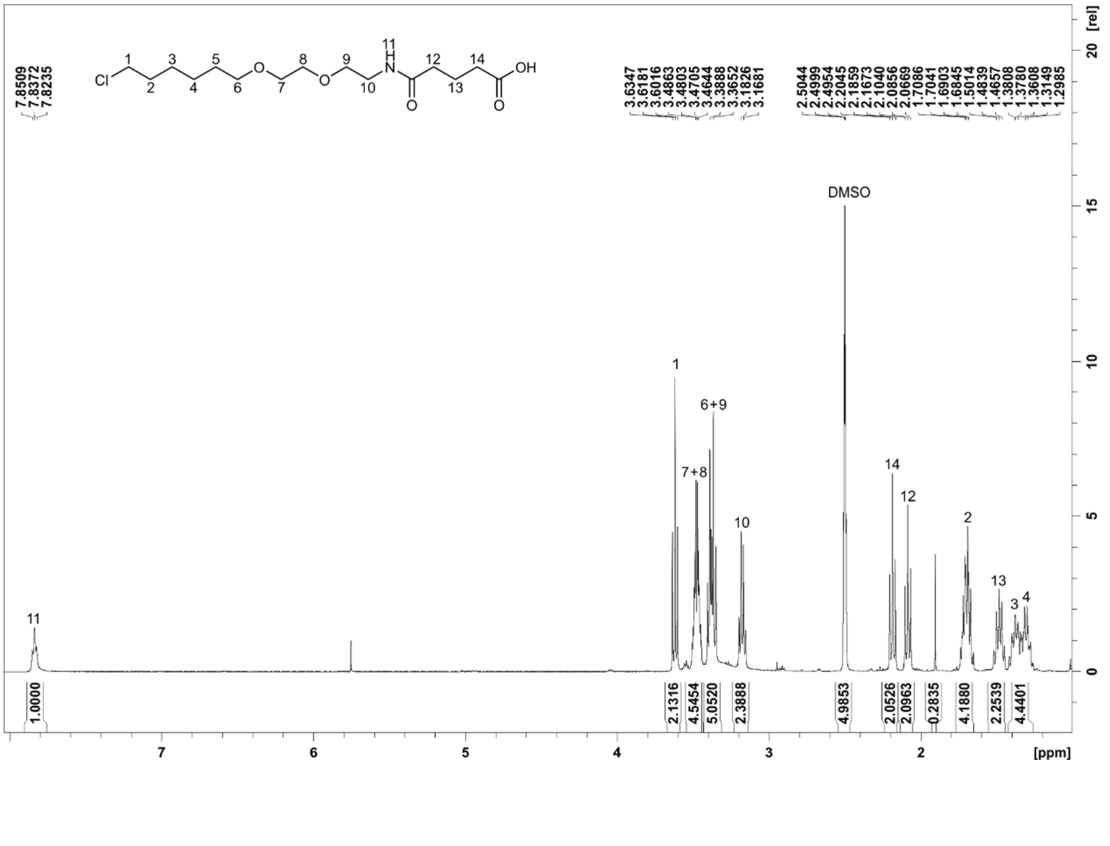

^{1}H NMR CA-GLA-NHS (6)

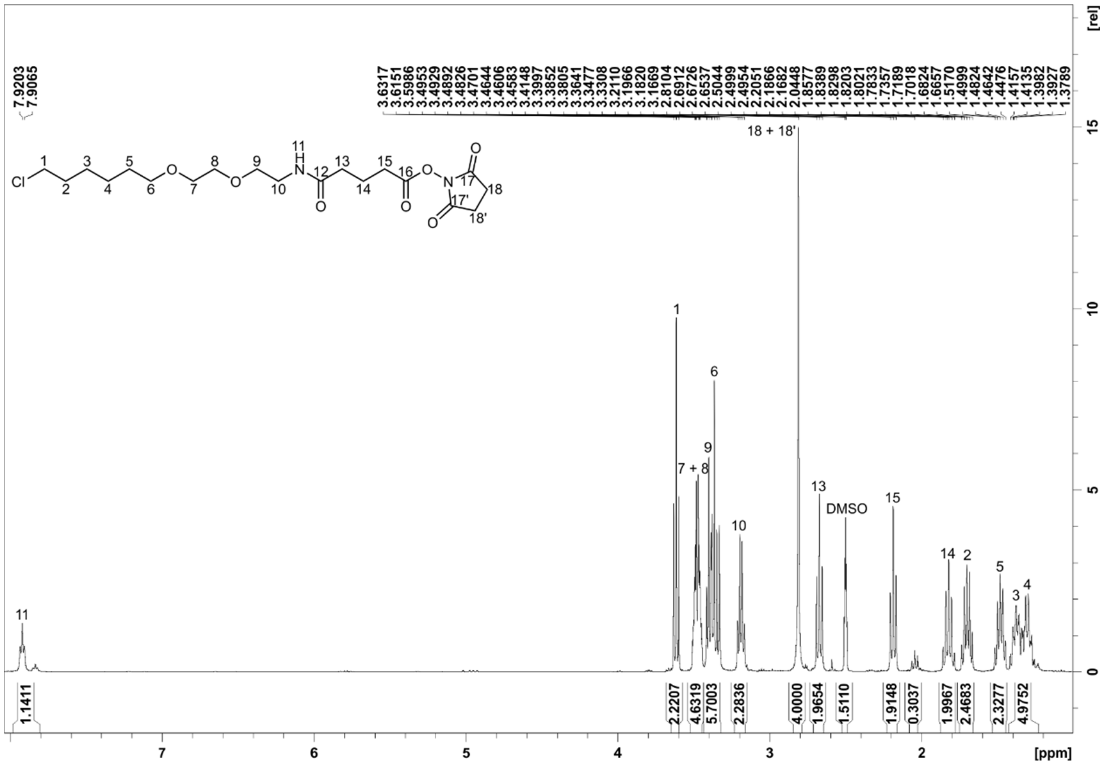

^{13}C NMR CA-GLA-NHS (6)

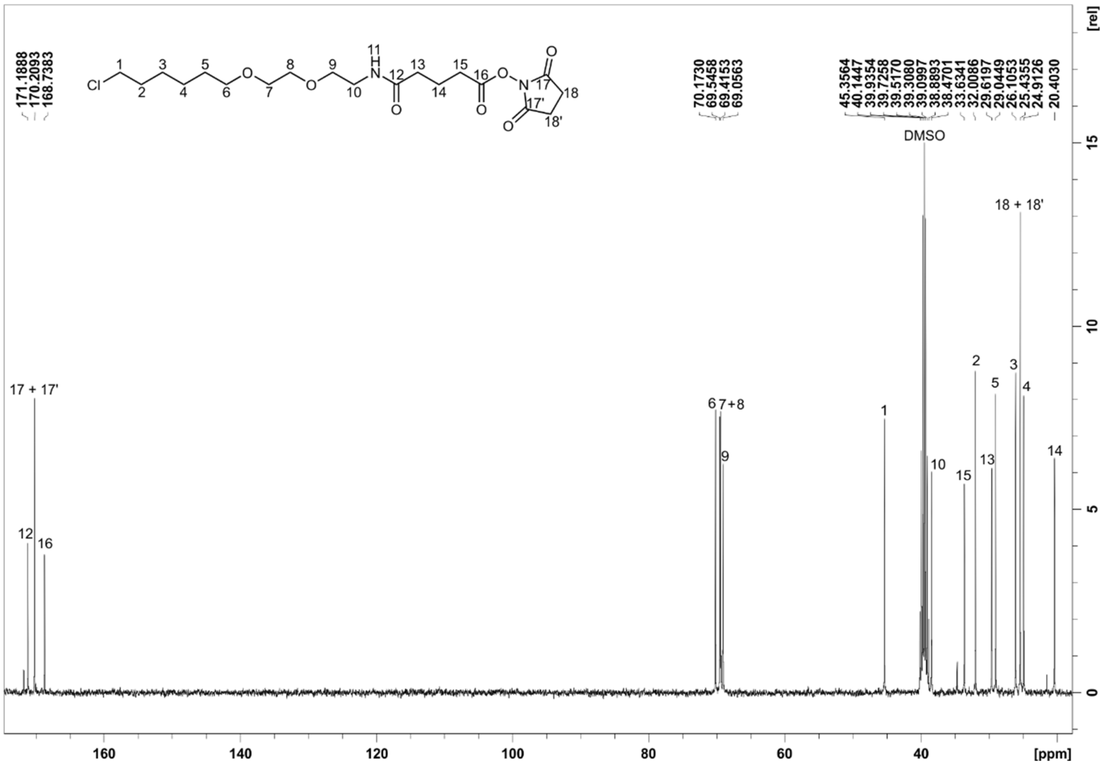

^{1}H-^{13}C HSQC CA-GLA-NHS (6)

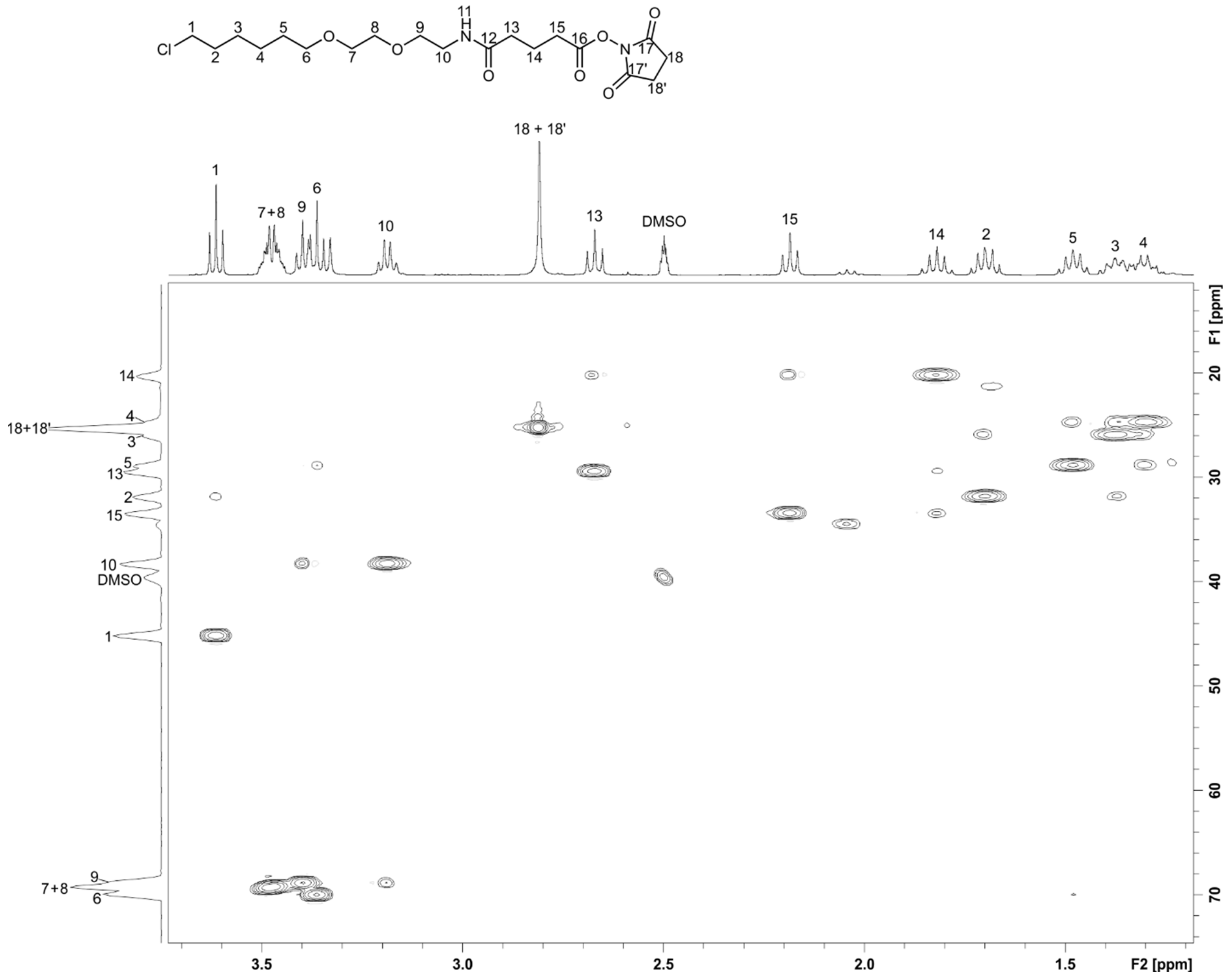

Uncropped PAGE images for Figure 3b

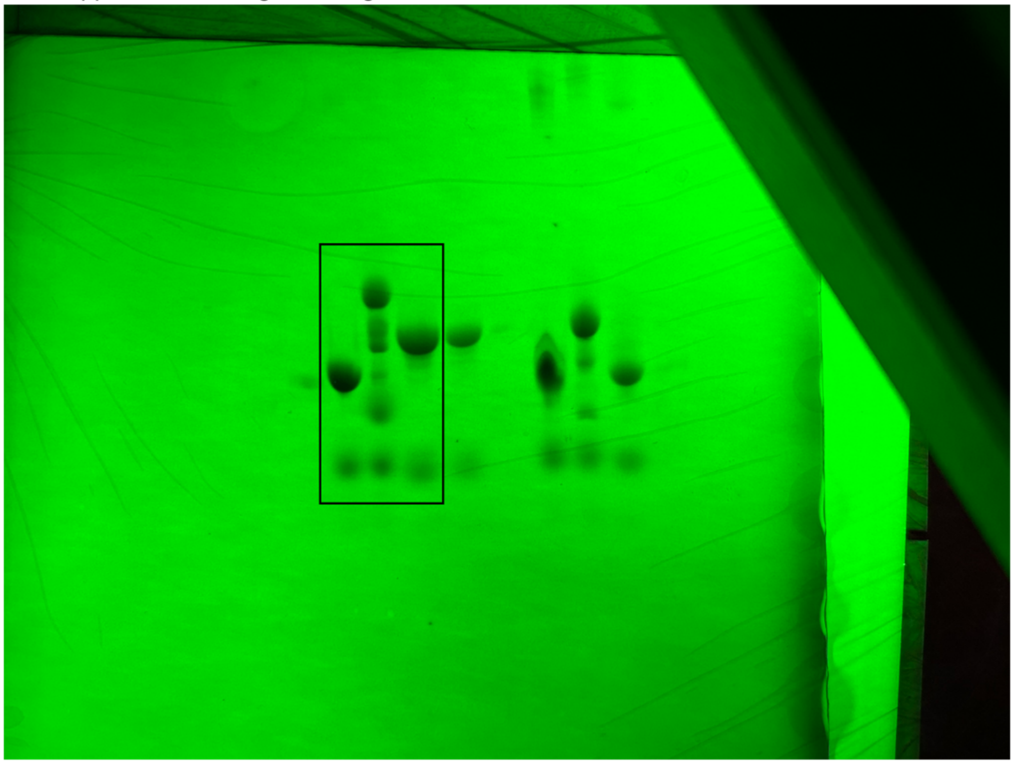

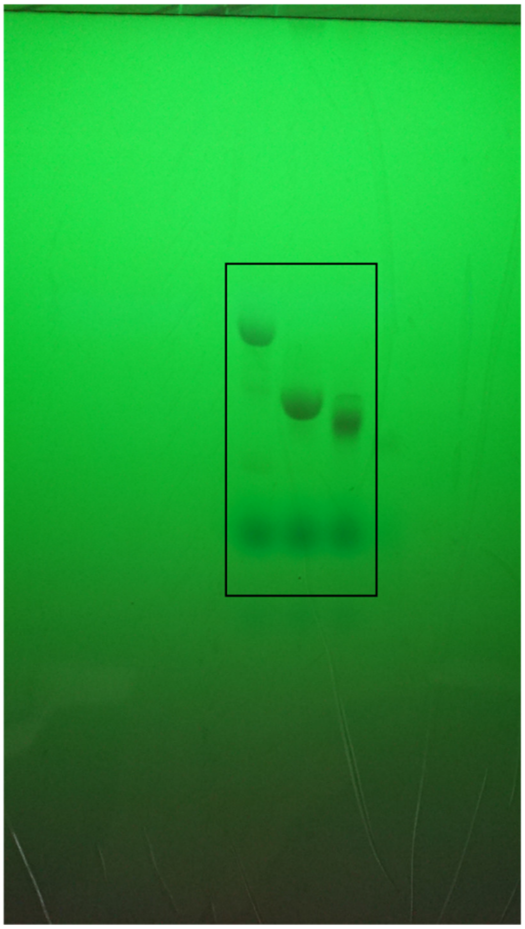

Supplement: Supplementary file 1 [file molecules-30-01049-s001.zip › molecules-3404812-supplementary.pdf]
